# Machine Learning and Non-Invasive Monitoring Technologies for Training Load Management in Women’s Volleyball: A Scoping Review

**DOI:** 10.3390/sports14020074

**Published:** 2026-02-07

**Authors:** Héctor Gabriel Sanhueza Tapia, Frano Giakoni-Ramírez, Josivaldo de Souza-Lima, Arturo Diaz Suarez

**Affiliations:** 1 Department of Physical Activity and Sport, University of Murcia, 30100 Murcia, Spain; ardiaz@um.es; 2 Faculty of Education and Social Sciences, Universidad Andres Bello, Las Condes, Santiago 7550000, Chile; frano.giakoni@unab.cl (F.G.-R.); josivaldo.desouza@unab.cl (J.d.S.-L.); 3 Faculty of Education Sciences, University of Granada, Prof. Vicente Callao Street, Beiro, 18011 Granada, Spain

**Keywords:** women’s volleyball, artificial intelligence, non-invasive monitoring, training load management, neuromuscular fatigue

## Abstract

Training load monitoring in women’s volleyball is a challenge for optimizing performance and mitigating injury risk. Non-invasive monitoring technologies and machine learning (ML) can support decision-making, but the evidence remains heterogeneous. This scoping review mapped and integrated the evidence on training load management, fatigue, and performance in women’s volleyball and identified gaps. The PRISMA Extension for Scoping Reviews (PRISMA-ScR) and the Joanna Briggs Institute (JBI) framework were followed. A systematic search was conducted in Scopus, Web of Science, and PubMed, covering January 2020 to September 2025. We included studies in female players at any competitive level, including mixed-sex studies meeting a minimum threshold of female participation, that evaluated external and/or internal load, neuromuscular or perceptual fatigue, and/or performance, using standardized data extraction and narrative/thematic synthesis. Fifty-three studies were included. Inertial measurement units (IMUs), force platforms, heart rate (HR) and heart rate variability (HRV), wellness questionnaires, and global/local positioning systems (GPSs/LPSs) were most prevalent. External-load intensity indicators (e.g., high-intensity jumps and accelerations) were reported as more sensitive to fatigue-related changes than accumulated volume. Machine learning models were less frequent and were mainly applied to multi-source integration and fatigue/readiness prediction, with recurring limitations in external validation and interpretability. Women-specific biological moderators, such as the menstrual cycle, were rarely addressed.

## 1. Introduction

Performance optimization has become progressively more demanding and complex, as competitive outcomes emerge from the dynamic interaction among physical, technical–tactical, and psychological dimensions throughout the training process and the competitive calendar, structured through periodization [1]. This perspective is reinforced by recent evidence conceptualizing athletes as a complex adaptive system, in which performance determinants interact in a non-linear, context-dependent, and individualized manner, requiring more integrated and operational monitoring approaches for decision-making [2].

In this scenario, integrated training-load monitoring is a cornerstone of contemporary planning. The combined quantification of external load (work performed: volume, intensity, density, and task structure) and internal load (psychophysiological response to the stimulus, including the session rating of perceived exertion; s-RPE) enables more precise modulation of adaptive processes and may mitigate fatigue or soft-tissue injury risk [3]. However, the applied usefulness of these systems depends on the feasibility of measurements in real-world settings and on outputs that support timely decisions. In this regard, Neumann et al. [2] emphasize the relevance of metrics and algorithms capable of operating in real or near-real time within a complex-systems framework, strengthening multidimensional monitoring for load control and data-informed decision-making.

The effectiveness of this approach is supported by the development of non-invasive tools that enable continuous quantification of relevant variables without interfering with training or competition processes, while preserving ecological validity in game-relevant contexts [4]. In recent years, wearable technologies have increased the capacity to simultaneously monitor internal and external parameters, facilitating faster and more individualized adjustments aimed at minimizing fatigue and optimizing adaptation [5]. In parallel, advances in data analytics have promoted evidence-based strategies for designing individualized programs that integrate physical, physiological, and psychological profiles, with the aim of optimizing performance and reducing injury risk [6].

At this point, it is crucial to distinguish between monitoring technologies and advanced analytical models. Machine learning (Machine Learning, ML) approaches and, more broadly, artificial intelligence (Artificial Intelligence, AI) are proposed as an analytical layer to integrate multi-source signals and identify complex relationships that may be overlooked by traditional statistical approaches [7]. To avoid conceptual ambiguity (and to ensure alignment between title, aim, and content), this review will consider AI/ML only those approaches that: (i) train a data-driven model to predict, classify, or detect patterns (supervised or unsupervised) and (ii) report an explicit validation procedure (e.g., train/test split or cross-validation) and/or performance metrics. In contrast, conventional descriptive or inferential analyses (e.g., correlations or group comparisons without predictive validation) will not be classified as AI/ML within this review framework [7]. This distinction is particularly relevant because, in applied literature, a substantial proportion of studies focus on monitoring technologies without explicitly incorporating AI/ML, which may create a perception of “misalignment” if AI/ML is not operationally defined.

Women’s volleyball has increased its competitiveness and expansion within team sports [8], intensifying demands on load management and rapid performance optimization [9,10]. Moreover, injury prevention through load-monitoring and load-adjustment strategies has become a central component of training support [11,12]. In this context, the convergence of non-invasive technologies and AI/ML models offers an opportunity to integrate information associated with external and internal load with applied orientation; however, the available evidence remains heterogeneous in technologies, outcomes, and analytical approaches, and does not consistently incorporate women-specific biological moderators in a systematic manner. Therefore, a scoping review is warranted to map the state of the evidence, clarify what is being measured and how it is analyzed, and delineate priority gaps for research and implementation.

To improve clarity and applicability, this scoping review is organized into three complementary domains (pillars), whose added value is made explicit relative to classical approaches to load and fatigue.

### 1.1. Comprehensive Characterization of Responses to Training Load

This pillar extends the traditional internal–external load approach by prioritizing integrated stimulus–response profiling in real-world settings, rather than focusing solely on the comparison between planned and executed load, a relationship that remains insufficiently documented in women’s volleyball. Its added value lies in describing adaptation patterns and “discordances” between metrics (e.g., high external load with low internal load, or vice versa), which are relevant for individualized prescription adjustments. The relevance of integrating load and well-being is illustrated by Kupperman et al. [3], who report complex seasonal patterns that are unlikely to emerge from isolated indicators. Similarly, Tometz et al. [13] demonstrate the feasibility of simultaneously monitoring internal and external variables during competition to characterize real match demands, while Bouzigues et al. [14] highlight that modeling performance based on jump load and strength-training characteristics can identify external-load components linked to in-match performance.

### 1.2. Multidimensional Fatigue Assessment

This pillar conceptualizes fatigue as a multifactorial phenomenon expressed across neuromuscular, physiological/autonomic, and perceptual domains. Its added value consists of promoting an operational triangulation of measures (rather than substituting one for another) to more accurately interpret recovery and readiness to perform. At the neuromuscular level, Setuain et al. [15] describe relevant changes in jump performance following systematic training sessions. Complementarily, Marzano-Felisatti et al. [16] provide algorithmic developments that increase the sensitivity of neuromuscular monitoring related to jump performance. Within the physiological domain, heart rate variability (HRV) is consolidated as a biomarker of autonomic status and internal load in volleyball [17]. Integrating HRV with perceptual metrics (well-being and s-RPE) enables a more precise reading of the stress–recovery balance to support daily decisions on jump exposure, intensity distribution, and recovery.

### 1.3. Application of AI/ML Models and Predictive Tools

This pillar addresses the technological frontier by incorporating AI/ML as a decision-support layer, explicitly differentiating it from instrumentation alone. Its added value is not to “replace” expert judgment, but to provide multi-source integration and predictive estimates (e.g., fatigue/readiness) when the volume and variety of data exceed manual interpretation. The literature reports advances toward individualized ML approaches for monitoring fatigue and performance-related risk, as well as the development of algorithms for jump categorization using inertial measurement units (IMUs) and digitized networks for the analysis of sport-specific actions [17]. Additionally, ML models have been applied to quantify stress load and characterize key performance factors in adolescent athletes [14]. Nevertheless, for applied adoption it is critical to explicitly address recurring limitations (external validation and interpretability); therefore, this pillar incorporates these constraints as part of the evidence mapping.

Together, these three pillars structure a clearer and more transferable framework for understanding how non-invasive technologies and AI/ML approaches have been used in women’s volleyball. Finally, it is acknowledged that biases and domain-specific gaps persist in the development and application of wearable technologies in athletes, reinforcing the need to deepen the evidence base in this field and standardize reporting to improve applied utility [18].

To the best of our knowledge, there is no scoping review specifically focused on women’s volleyball that integratively maps the combined use of non-invasive monitoring technologies and AI/ML approaches to support training load and fatigue-related decision-making.

## 2. Materials and Methods

The present investigation corresponded to a scoping review, conducted in accordance with the methodological guidelines established in the JBI Manual for Evidence Synthesis [19] and reported following the PRISMA-ScR guideline. The completed PRISMA-ScR checklist is provided in Supplementary File S1. Protocol and registration: The review protocol was registered retrospectively in OSF Registries (DOI: 10.17605/OSF.IO/2FDUA; registered on 22 January 2026). This approach allowed for the systematic characterization and integration of the available scientific evidence on the application of AI models and/or non-invasive monitoring technologies in training load control in women’s volleyball, identifying key concepts, types of existing evidence, and gaps in current knowledge.

In accordance with PRISMA-ScR recommendations, evidence synthesis was conducted using a narrative and thematic approach, without statistical aggregation. The studies were grouped into three predefined analytical domains: (1) comprehensive characterization of responses to training load, (2) multidimensional assessment of fatigue, and (3) application of AI models and predictive tools.

These methodological pillars addressed the need to understand the athlete as a stress-responsive and adaptive system, in which multiple factors interact dynamically and in an individualized manner, determining responses to training and competitive performance [1]. The quantitative results reported correspond exclusively to findings from primary studies and were presented for descriptive purposes only, as representative examples of the evidence mapping.

Research Question

What non-invasive monitoring technologies and what machine learning approaches have been used in women’s volleyball to quantify external and internal load, monitor fatigue and/or estimate performance readiness states, and what methodological gaps limit their translation into practice

The PCC framework (Population–Concept–Context) used to operationalize eligibility criteria is summarized in Table 1.

### 2.1. Search Strategy

A systematic search of the scientific literature was conducted between January 2020 and September 2025 across the Scopus, Web of Science, and PubMed databases, following methodological guidelines for a scoping review. No systematic search of gray literature or preprints was performed. The process was carried out independently by two researchers in order to maximize search sensitivity and reduce selection bias. Additionally, the reference lists of the included articles were examined to identify relevant literature that may not have been retrieved through the primary search procedures. The complete search strategy for Scopus, Web of Science, and PubMed is provided in Supplementary Table S1.

### 2.2. Inclusion Criteria

Eligibility criteria were defined a priori in accordance with the PRISMA Extension for Scoping Reviews (PRISMA-ScR) and the methodological framework of the Joanna Briggs Institute (JBI). Studies were included if they simultaneously met the following requirements:

1. Population. Female volleyball athletes at any competitive level (competitive amateur, school, regional, university, national, semi-professional, and professional). Mixed-sex studies (women and men) were considered when they met a predefined minimum threshold of female participation (≥35%) and/or reported analyses or results specific to women. The ≥35% threshold was adopted for an operational purpose, aiming to balance the inclusivity required in an emerging field (where part of the evidence derives from mixed samples) with external validity for women’s volleyball. We acknowledge that when results are not disaggregated by sex, direct inference to women may be limited; therefore, this criterion is interpreted as an eligibility condition (not a guarantee of female specificity), and its implications are explicitly addressed in the interpretation of findings and in the limitations section.
2. Study design. We included: (i) observational studies (prospective/retrospective cohorts, cross-sectional, and longitudinal); (ii) descriptive studies; (iii) technological validation and/or accuracy studies; (iv) algorithm development, training, and evaluation studies; (v) experimental studies (randomized controlled trials and quasi-experimental designs); (vi) pilot and feasibility studies; (vii) case studies with methodological relevance; and (viii) systematic reviews, when they provided evidence directly linked to technologies or analytical approaches applicable to volleyball and contributed to mapping the field. Considering the diversity of designs included in a scoping review, studies were categorized by design type (e.g., technological validation/accuracy, observational, experimental/pilot/feasibility, and reviews) for synthesis and presentation purposes, avoiding treating them as equivalent in interpretive terms.
3. Phenomenon of interest/outcomes. We included studies that assessed at least one of the following domains: external load, internal load, neuromuscular fatigue, perceptual fatigue/well-being, performance readiness (readiness), performance, and/or injury prevention, in training and/or competition contexts in volleyball.
4. Technological exposure and/or analytical approach. We included studies that applied or evaluated: (a) Artificial intelligence/machine learning (AI/ML) only. Artificial intelligence and/or machine learning (AI/ML) models applied to volleyball-related data for classification, prediction, or pattern detection in load, fatigue, readiness, performance, or injury, provided that they reported an explicit training and validation procedure (e.g., train/test split or cross-validation) and/or performance metrics. Conventional descriptive or inferential analyses without predictive validation were not classified as AI/ML in this review. (b) Non-invasive monitoring technologies only. Non-invasive devices, sensors, or measurement systems to quantify load, assess multidimensional fatigue, or monitor readiness (e.g., inertial measurement units, force platforms, heart rate and heart rate variability, wellness questionnaires, global/local positioning systems), regardless of whether the analysis was descriptive or based on traditional statistical methods. (c) AI/ML + monitoring combination. Integrated systems combining non-invasive technologies with AI/ML models (as per the operational definition above) for multi-source integration and/or predictive estimation of states (e.g., fatigue/readiness). (d) Comparative studies. Studies comparing technologies, metrics, or analytical approaches (including AI/ML versus traditional approaches) regarding their practical value for monitoring load/fatigue/readiness and/or their association with performance or injury outcomes.

### 2.3. Exclusion Criteria

Studies were excluded if they met any of the following criteria: (a) Investigations that analyzed an exclusively male population, with no data applicable or transferable to women’s volleyball. (b) Articles that employed exclusively invasive methods. (c) Studies focused solely on post-injury clinical rehabilitation and not related to training load control in functionally active athletes. (d) Narrative reviews and editorials, although these were used exclusively for manual reference searching. (e) Research conducted in completely different sports disciplines, with no methodological or technological transferability. (f) Publications prior to the year 2020. (g) Studies published in languages other than Spanish, English, Portuguese, or Italian. (h) Purely theoretical articles without an empirical component or validation process, as well as brief communications lacking sufficient methodological detail. (i) Articles for which full texts could not be retrieved due to paywall restrictions, were unavailable through institutional access, and were not provided by the authors after two contact attempts separated by a fourteen-day interval.

### 2.4. Study Selection Process

The study selection process was conducted in accordance with the PRISMA Extension for Scoping Reviews (PRISMA-ScR) and the methodological recommendations of the Joanna Briggs Institute (JBI). The search strategy was implemented in Scopus, Web of Science, and PubMed due to their complementarity and broad interdisciplinary coverage: Scopus and Web of Science capture literature across sport sciences, engineering/technology, and behavioral sciences with extensive journal indexing, whereas PubMed provides greater sensitivity in biomedical and physiological domains relevant to internal load, fatigue, and injury. No study-design restrictions were applied during the identification phase, consistent with the purpose of a scoping review, and the process was complemented by a subsequent PCC-based screening stage.

Regarding language, priority was given to literature that could be rigorously assessed by the review team, considering that English represents the majority of indexed scientific output in sport sciences and applied technology. However, an inclusive approach was maintained during screening by evaluating all retrieved records, and restrictions were applied only when full-text assessment was not methodologically reliable due to language barriers or access limitations. This approach aims to reduce language bias, although it is acknowledged as a latent limitation in evidence synthesis.

During the identification stage, 339 records were retrieved from Scopus (*n* = 148), Web of Science (*n* = 145), and PubMed (*n* = 46). Subsequently, 168 duplicates were removed using automated and manual procedures in Mendeley, and the de-duplicated database was migrated to Rayyan to ensure traceability and consistency during screening. In the screening phase, two investigators independently assessed titles and abstracts of the remaining 171 records using PCC-based inclusion criteria; 76 records were excluded for not meeting the protocol criteria.

For full-text assessment, 95 reports were considered. At this stage, 30 articles were excluded for methodological or relevance reasons, including: absence of sex-disaggregated data or a female sample <35% without applicable analysis; studies focused exclusively on clinical rehabilitation unrelated to load management; lack of an empirical component or insufficient methodological validation for mapping purposes; lack of relevance to load control, fatigue, or performance in volleyball; and ineligible study designs according to the protocol. Additionally, 12 articles were excluded due to restricted access to the full text.

Regarding access-related bias, we acknowledge that excluding unavailable full texts may introduce selection bias (availability/access bias), potentially overrepresenting evidence from more accessible journals (e.g., open access or journals covered by institutional subscriptions) and underrepresenting findings from certain regions, publishers, or technological lines. Therefore, the evidence map may be partially shaped by availability rather than scientific scope alone. To mitigate this risk, the Limitations section specifies that findings should be interpreted as a map of accessible and assessable evidence, and that future updates may incorporate additional retrieval strategies (e.g., contacting authors, interlibrary loans) where feasible.

Finally, 53 studies met the eligibility criteria and were included in the synthesis. Data were stored in an editable structured extraction matrix including: author, title, country, sample, age range, monitoring technology and/or AI/ML approach, and main outcomes related to load, fatigue, performance, or injury. In line with editorial recommendations for transparency and reproducibility, this matrix is provided in Supplementary Table S2.

Consistent with PRISMA-ScR and JBI guidance for scoping reviews, we did not conduct a formal critical appraisal (risk of bias/quality assessment) of the included sources because the purpose of this review was to map and characterize the available evidence rather than to estimate effects or determine comparative effectiveness. Therefore, findings should be interpreted as descriptive and hypothesis-generating.

**Figure 1:**
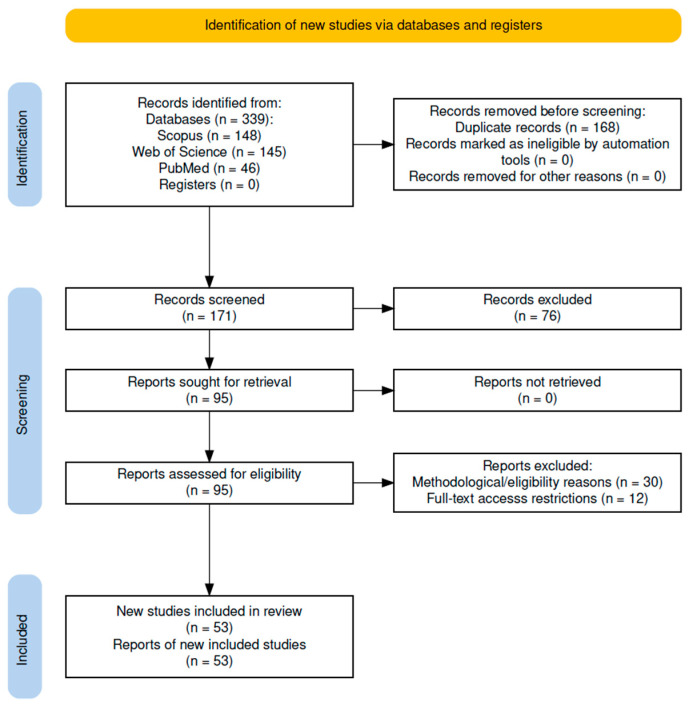
PRISMA-ScR flow diagram of the study selection process for the scoping review.

The PRISMA-ScR flow diagram of the study selection process is presented in Figure 1.

Of the 53 included sources, 48 were primary empirical studies and five were secondary syntheses (scoping/systematic reviews). Within primary studies, 38 (79.2%) involved women only samples, nine (18.8%) included mixed sex samples meeting the ≥35% female eligibility criterion, and one (2.1%) involved a male-only volleyball cohort retained for methodological/technological transferability without informing women-specific inferences. Importantly, among mixed-sex primary studies, six of nine (66.7%) reported sex-stratified analyses or explicitly female-specific outcomes, whereas three of nine (33.3%) did not provide sex disaggregated results and were therefore interpreted cautiously and used primarily for contextual evidence mapping. When the narrative interpretation was restricted to women only and sex stratified evidence (n = 44/48 primary studies, 91.7%), the main technological patterns and evidence gaps remained unchanged.

## 3. Results

Given the scoping review design, no meta-analysis or pooled effect size estimation was performed. All reported statistical values correspond to individual primary studies and are used solely to illustrate ranges and recurring patterns within the evidence mapping.

To avoid conceptual ambiguity and to address the distinction between monitoring technologies and artificial intelligence/machine learning (AI/ML) models, studies were coded and presented in three analytical categories: (i) non-invasive monitoring technologies without a predictive layer (monitoring-only), (ii) AI/ML-based approaches (AI/ML-only), operationally defined as data-driven models trained for classification, prediction, or pattern detection that report an explicit validation procedure (e.g., train/test split or cross-validation) and/or performance metrics, and (iii) combined systems (AI/ML + monitoring). Accordingly, instrumentation/monitoring findings are synthesized in Section 3.1, Section 3.2, Section 3.3, Section 3.4 and Section 3.5, whereas evidence meeting the operational AI/ML definition is presented separately in Section 3.6. Conventional descriptive or inferential analyses without predictive validation are not classified as AI/ML within this review.

The general characteristics of the evidence are summarized in Table 2, while the synthesis of technologies, metrics, and main patterns is presented in Table 3 and Table 4. The evidence comprised 53 studies conducted across 23 countries, with populations ranging from youth to professional levels, and ages spanning from 12.5 years [20] to 51.1 years in veteran athletes [21]. Sample sizes varied widely, from pilot studies with small samples (n = 3–6) [22] to longitudinal investigations including up to 125 athletes [17]. Regarding modality, indoor volleyball predominated (n = 45; 83.3%) over beach volleyball (n = 9; 16.7%), consistent with previous reports on data availability [23,24].

**Table 2:** Overview of the included evidence.

Domain	Findings (Evidence Map)
Scope	23 countries; evidence ranging from youth to professional levels
Age range (examples)	12.5 years [20] to 51.1 years [21]
Sample size (examples)	Pilot studies n = 3–6 [22] to longitudinal cohorts up to n = 125 [17]
Modality	Indoor n = 45 (83.3%); Beach n = 9 (16.7%) [23,24]
Predominant study designs	Longitudinal observational studies; validation/accuracy studies; pilot/experimental studies; systematic reviews (for mapping)
Primary focus	External and internal load; neuromuscular/perceptual fatigue; performance and/or injury risk

### 3.1. Predominant Technologies and Metrics Used

In this subsection, non-invasive monitoring technologies and metrics are reported. When studies used signal-processing algorithms, calibration procedures, or rule-based classification for instrumental purposes, these were considered part of instrumentation/monitoring, unless they involved AI/ML models trained with explicit validation and performance metrics (presented in Section 3.6).

Across the included studies, inertial measurement unit (IMU)-based systems were the most frequently used technology, with the VERT device recurrently reported [3,25,26,27]. From an applied perspective, IMUs were primarily used to quantify external load and weekly training demands [28,29]. Validation evidence supported high agreement for jump count against expert observation (95–99%) and good agreement with reference standards [6], whereas jump-height estimation showed greater device- and algorithm-dependent variability, with systematic deviations reported across systems [30,31]. In competitive contexts, the WIMU PRO system was reported as valid [16], and additional evidence addressed internal-load quantification in similar game scenarios [32].

Force platforms, typically using the countermovement jump (CMJ), enabled the analysis of multiple force–time metrics [33], with high reliability for jump height (intraclass correlation coefficients, ICC > 0.90) in validation studies [31]. Mechanical changes in CMJ performance associated with increased competitive load or seasonal variations were described and proposed as useful neuromuscular markers for applied monitoring [34]. The evidence also underscored the need for sex-specific reference curves and thresholds to interpret risk and performance [35]. Notably, despite the specific focus on women, biological moderators relevant to women’s volleyball (e.g., menstrual-cycle-related variables) were infrequently incorporated in primary-study designs and reporting, constituting a cross-cutting gap with direct implications for individualized monitoring.

Regarding positioning technologies, GPSs were reported mainly in beach volleyball [24,25,26,27,28,29,30,31,32,33,34,35,36], whereas local positioning systems (LPS) were applied in indoor contexts [10,11,12,13,14,15,16,17,18,19,20,21,22,23,24,25,26,27,28,29,30,31,32,33,34,35,36,37]. These metrics were often interpreted alongside weekly variations in internal/external load, reinforcing their utility for supporting decision-making when intensity fluctuated [38].

Heart rate (HR) and heart rate variability (HRV) monitoring—commonly using validated Polar systems—were used as indicators of internal load and autonomic status, providing reference values to support comparability [39]. Evidence syntheses and studies on wearable technologies and biomechanical assessment reinforced the role of these systems within contemporary volleyball load-monitoring frameworks [8,18]. A condensed view of technologies, typical outputs, and applied uses is presented in Table 3.

**Table 3:** Predominant non-invasive technologies and typical metrics in women’s volleyball.

Technology	Typical Metrics/Outputs	Primary Applied Use	Representative Evidence
IMU (incl. VERT)	Jump count; accelerometry-derived load; algorithm-dependent jump height	External load; weekly monitoring; jump profiling	Recurrent VERT use [3,25,26,27]; weekly quantification [28,29]; jump-count validity [6]; jump-height limitations [30,31]; WIMU PRO in competition [16]
Force platforms (CMJ)	Jump height; force–time metrics; neuromuscular proxies	Fatigue/readiness; seasonal neuromuscular profiling	CMJ and multiple metrics [33]; reliability [31]; changes with competitive volume [34]; sex-specific references [35]
GPS/LPS	Distance; movement patterns; acceleration/deceleration exposure	Contextualizing demands (beach vs. indoor; role)	GPS in beach volleyball [24,25,26,27,28,29,30,31,32,33,34,35,36]; LPS in indoor volleyball [10,11,12,13,14,15,16,17,18,19,20,21,22,23,24,25,26,27,28,29,30,31,32,33,34,35,36,37]; weekly variations by intensity [38]
HR/HRV	Heart rate (HR); heart rate variability (HRV; e.g., rMSSD)	Internal load; autonomic status/recovery	Comparable benchmarks [39]; support within wearable frameworks [8,18]
Perceptual tools	Session rating of perceived exertion (s-RPE); wellness; Total Quality Recovery (TQR)	Low-cost internal monitoring; recovery	s-RPE and associations [13]; tactical-context limitations [40]; TQR associated with HRV/stress [41]

### 3.2. External and Internal Load Demands by Training and Competition Context

Across several studies, training sessions accumulated higher accelerometry-derived loads than individual matches, which was attributed to longer exposure time and the rotational structure of play [3]. Nevertheless, in competitive settings, total load could exceed that typically observed in training sessions [28]. It was also noted that perceived load may be influenced by contextual stressors (e.g., academic demands), supporting the need for multifactorial monitoring to interpret athletes’ adaptations [42].

In beach volleyball, higher external demands than in indoor volleyball were reported, including increases in relative distance and explosive kinematic load (PlayerLoad), largely attributable to surface instability [37]. Complementary biomechanical evidence showed kinetic differences in jumps performed on sand versus rigid surfaces, underscoring the importance of context/surface when prescribing training and interpreting monitoring metrics [43]. Competitive level also emerged as a moderator of psychophysiological responses [44], and in multi-day tournaments involving youth populations, relatively stable daily loads were reported without clear cumulative deterioration, consistent with adequate overnight recovery in those scenarios [25]. These context-dependent patterns are summarized in Table 4.

### 3.3. Position-Specific Differences and Load Profiles

Consistent position-related patterns were reported: middle blockers accumulated a higher volume of high-intensity jumps, setters performed the highest total number of jumps, and defensive roles exhibited greater multidirectional displacement demands [45]. These findings aligned with evidence linking technical–tactical demands to specific external-load profiles [3] and with studies reporting high between-position variability in jump profiles and technical constraints [46]. In beach volleyball, role specialization also differentiated acceleration/deceleration and jump profiles between defenders and blockers [36]. The position-specific synthesis is integrated in Table 4.

### 3.4. Neuromuscular Fatigue, Sensitive Metrics, and Predictive Relationships

Across studies using force platforms and countermovement jump (CMJ) profiling, it was repeatedly highlighted that metrics reflecting stimulus intensity may be more sensitive to fatigue-related changes than indicators based solely on volume [33]. Jump variables were also associated with task-specific performance (e.g., attack jump performance), reinforcing the need to quantify jump load with sufficient precision [47]. Beyond jump counts, total accelerations were reported as a better predictor of perceived fatigue (rating of perceived exertion/session rating of perceived exertion; RPE/s-RPE) than jump volume, emphasizing the importance of monitoring multidirectional demands [29]. Similarly, internal-load measures were linked to next-day fatigue/perceptual responses in different reports [48,49].

Load-modeling approaches such as the acute:chronic workload ratio (ACWR) and exponential weighted moving average (EWMA) methods were applied to detect in-season load spikes [50]. From an autonomic perspective, heart rate variability (HRV) indices such as the root mean square of successive differences (rMSSD) were reported as sensitive to recovery and load changes [41], and seated assessments showed correspondence with cardiovascular remodeling markers compared with supine evaluations [51]. Considerations of mental fatigue were also incorporated, suggesting that perceptual indicators may be influenced by cognitive/tactical demands [52]. Post-competition CMJ decrements were described as fatigue markers and discussed in relation to risk contexts (e.g., anterior cruciate ligament) [53], while reliability evidence supported the CMJ as a monitoring tool [31]. Session rating of perceived exertion (s-RPE) showed robust associations with physiological methods (training impulse; TRIMP) and external indicators (distance), supporting its practical utility [13], although limitations during tactical/competitive sessions were reported in some populations [40]. The Total Quality Recovery (TQR) scale was also associated with HRV and stress, supporting its feasibility for monitoring recovery [41]. These findings are detailed in Table 4.

### 3.5. Injuries, Load Variability, and Biomechanical Risk Indicators

In injury-related evidence, greater load variability was associated with higher injury incidence, and injured athletes showed distinct exposure patterns in the periods preceding injury [27]. Relevant neuromuscular/biomechanical risk markers were highlighted, including asymmetries in unilateral jump performance and landing-related signals [54]. Complementary studies identified dynamic knee valgus, ankle mobility, foot stability, and strength profiles as relevant components for interpreting risk and jump performance [55,56,57]. Comparisons between bilateral and unilateral jumps suggested that they provide complementary, non-interchangeable information on take-off and landing asymmetries [58]. Applied strategies such as post-activation performance enhancement (PAPE) were also reported, with specific time windows for optimization [59]. In beach volleyball, relevant decrements were not always observed when comparing 24 h versus 48 h recovery windows in trained athletes [60]. Competitive level was additionally described as a moderator of neuromuscular responses to stress stimuli [46]. Injury/risk patterns are summarized in Table 4.

### 3.6. Artificial Intelligence/Machine Learning, Non-Linear Models, and Applied Tolos

This subsection includes only studies that met the operational definition of AI/ML adopted in this review (model training + explicit validation and/or performance metrics). Studies focused on monitoring technologies using conventional statistical analyses are synthesized in Section 3.1, Section 3.2, Section 3.3, Section 3.4 and Section 3.5. Within the mapped evidence, ML applications were less frequent than monitoring-only studies; when implemented, they were mainly oriented toward multi-source integration and the prediction/estimation of functional states such as fatigue/readiness or performance-related outcomes.

Across the available evidence, non-linear models were reported as showing superior predictive performance compared with linear approaches in specific datasets, with algorithms such as Random Forest being used to model non-linear relationships between load, exposure, and performance outcomes. These models identified predictors primarily linked to workload and exposure to high-intensity actions [14]. However, AI/ML studies showed recurring limitations that constrain applied transferability, particularly regarding external validation, generalizability across contexts, and interpretability requirements for real-world decision-making in the field.

Beyond prediction, applied-oriented developments were reported using real-time biofeedback systems that integrated biomechanical variables to optimize landing patterns and facilitate transfer to sport-specific tasks, illustrating the potential of computational systems to support motor-learning processes under controlled settings [61]. Emerging applications involving large language models (LLMs) to generate training programs in women were also described, showing potential improvements in jump-related variables; however, findings were heterogeneous and do not support systematic replacement of expert-designed programming. Instead, they suggest a complementary role for these tools when used with professional oversight and appropriate validation criteria [62].

Overall, this evidence indicates that the AI/ML component within the field remains less prevalent than instrumental monitoring, and that its current contribution is mainly focused on information integration and predictive/decision-support applications, with methodological gaps that should be considered when interpreting its practical applicability (see Table 4).

**Table 4:** Main patterns, trends, and gaps: narrative/thematic synthesis.

Theme/Pattern	What the Evidence Consistently Suggests (Mapping)	Representative Evidence
Intensity > volume for fatigue sensitivity	Exposure to high-intensity actions (e.g., very high jumps and multidirectional acceleration demands) is more frequently reported as being associated with fatigue-sensitive outcomes than accumulated volume alone; supports monitoring the “quality” of exposure alongside quantity, within the descriptive scope of a scoping review.	Force–time associations [33]; accelerations vs. RPE/s-RPE [29]
Context moderates load (training/match; indoor/beach; surface)	Training sessions may accumulate higher accelerometric load than a single match due to longer exposure and the rotational structure of play; however, competition can exceed typical training load in specific scenarios. Beach volleyball generally shows higher external demands, plausibly linked to the surface, requiring context- and modality-specific interpretation.	Training vs. match [3,28]; beach vs. indoor [37]; surface-related kinetics [43]
Position/role differences	Load profiles differ systematically by position/role (jump exposure vs. multidirectional displacement); therefore, team-level averages can mask meaningful between-position variability and reduce applied interpretability.	Position patterns [45]; multidirectional displacement [3]; positional variability [46]; beach roles [36]
Multimodal monitoring improves interpretability	Integrating external load with autonomic (HRV) and perceptual (s-RPE/TQR/wellness) indicators improves interpretation of the stress–recovery balance and reduces single-metric misinterpretation; perceptual sensitivity may vary with cognitive/tactical demands, so context-aware interpretation is required.	HRV rMSSD and recovery/load [41,51]; cognitive/perceptual influence [52]; s-RPE utility and limits [13,40]; TQR [41]
Injury/risk: variability and asymmetries	Greater load variability is associated with injury; asymmetries and landing-related markers provide useful risk signals. Bilateral and unilateral assessments offer complementary (non-interchangeable) information about take-off/landing asymmetries.	Variability and injuries [27]; asymmetries [54]; biomechanical markers [55,56,57]; bilateral vs. unilateral complementarity [58]
Women-specific biological moderators (cross-cutting gap)	Despite the women-focused scope, women-specific biological moderators (e.g., menstrual-cycle-related variables or other biological modulators) are incorporated infrequently and unsystematically, limiting transfer to truly individualized monitoring.	Calls for sex-specific reference curves/thresholds for metric and risk interpretation [35]
AI/ML: potential with validation and transfer barriers	AI/ML evidence is less prevalent than monitoring-only studies; it mainly targets multi-source integration and prediction/estimation (fatigue/readiness/performance). Applied adoption is constrained by recurring gaps in external validation, cross-context generalization, and interpretability/calibration requirements.	Non-linear models and prediction [14]; applied biofeedback [61]; LLMs as complementary tools [62]

## 4. Discussion

This scoping review mapped how non-invasive monitoring technologies and, less frequently, artificial intelligence/machine learning (AI/ML) approaches have been applied to training load management, fatigue monitoring, performance, and injury-related outcomes in women’s volleyball. Consistent with a scoping review design, this Discussion synthesizes the direction and recurrence of reported patterns and the methodological characteristics of the field, rather than estimating pooled effects or establishing comparative effectiveness.

To improve clarity and applied transferability, and in response to reviewer feedback, the interpretation is structured around (i) monitoring technologies and operational metrics, (ii) multidimensional fatigue and injury-related signals, and (iii) AI/ML applications, emphasizing definitional boundaries and the main constraints for implementation.

This mapping aligns with conceptual work describing sport performance as the product of interacting physical, technical–tactical, and psychological dimensions within a complex adaptive system, which motivates integrated monitoring and context-sensitive decision support [1,2].

Notably, despite the specific focus on women, biological moderators relevant to women’s volleyball (e.g., variables related to the menstrual cycle) were infrequently incorporated into the design and reporting of primary studies, representing a cross-cutting gap with direct implications for individualized monitoring.

### 4.1. Main Interpretation of the Findings

Across the mapped evidence, monitoring-only studies predominated. Inertial measurement units (IMUs) were the most frequently used technology, with VERT devices recurrently applied to quantify jump exposure and accelerometry-derived external-load indicators [3,23,24,25]. Validation studies generally supported high agreement for jump counts against expert observation [6,26], whereas jump-height estimation showed device- and algorithm-dependent error, limiting interchangeability across systems [24,27]. Force platforms (typically via countermovement jump testing) and heart rate/heart rate variability (HR/HRV) monitoring provided complementary internal and neuromuscular signals and were often used as reference measures or to triangulate fatigue and readiness states [10,29,30,31]. Additional evidence supported wearable deployment in competitive contexts and reinforced the relevance of biomechanical assessment and wearable monitoring as part of contemporary load-control frameworks [8,16,18].

Across the mapped primary studies, intensity-weighted external-load indicators (e.g., very high-intensity jumps and multidirectional acceleration demands) were more frequently reported as being associated with fatigue-sensitive outcomes than accumulated volume alone [15,27,34]. In several reports, exposure intensity showed more consistent relationships with subsequent neuromuscular changes than jump counts, which supports, from an applied perspective, monitoring the “quality” of exposure alongside its quantity, within the descriptive and hypothesis-generating scope of a scoping review [3,34].

Context also moderated load interpretation. Training sessions often accumulated greater accelerometric load than single matches due to longer exposure and the rotational structure of play [3], whereas competition could exceed typical training load in specific scenarios [28]. Overall, beach volleyball showed higher external demands than indoor volleyball, plausibly linked to the mechanical and energetic constraints of sand surfaces [37]. Complementary biomechanical evidence reported kinetic differences in jumping tasks performed on sand versus rigid surfaces, underscoring the importance of context and surface when interpreting jump-related metrics [43].

Position- and role-specific profiles were repeatedly reported, indicating that global team averages can mask meaningful between-position variation in jump exposure and multidirectional movement demands [45]. Accordingly, position-sensitive monitoring and reporting may improve interpretability, and evidence showed high variability in jump profiles and technical constraints across positions and competitive levels [46]. In beach volleyball, role specialization likewise differentiated acceleration/deceleration and jump profiles between defenders and blockers [36].

The mapped evidence also supports a multimodal monitoring rationale: external-load signals (IMU/GPS/LPS) become more interpretable when contextualized with internal-load and recovery indicators, such as session rating of perceived exertion (s-RPE), wellness measures, and heart rate variability (HRV) [13,41]. For example, rMSSD has been reported as sensitive to recovery and training-load changes within volleyball settings [17,39,41,51]. Perceptual indicators are highly feasible, but their sensitivity may vary with session content and cognitive/tactical demands, highlighting the need to interpret them within context [42,52]. In indoor volleyball contexts, local positioning systems (LPS) represent a promising avenue to quantify trajectories, speed, and exposure to high-intensity actions in confined spaces where GNSS/GPS is not feasible. Emerging implementations based on ultra-wideband (UWB) or Bluetooth Low Energy (BLE), potentially embedded within Internet of Things (IoT) architectures for real-time capture and analytics, could complement IMU-derived kinematics by improving the spatial contextualization of external load. This technological direction may enhance decision support for load management in indoor environments, although further volleyball, specific validation particularly in women’s cohorts and under ecologically valid training and match conditions is needed. In parallel, the limited incorporation of women-specific moderators remains a critical gap; beyond calls for sex-specific reference curves and thresholds for neuromuscular metrics and risk interpretation [35], variables related to the menstrual cycle or other biological moderators were rarely modeled, which restricts translation to individualized practice in women’s volleyball.

AI/ML studies were less common than monitoring-only studies and were mainly used for multi-source integration and prediction of fatigue/readiness states or performance outcomes. In the limited comparative evidence, non-linear approaches (e.g., Random Forest) showed higher predictive performance than linear models in specific datasets; however, these findings remain study-specific and require external validation and interpretable outputs to support applied adoption [14]. At the applied frontier, real-time biofeedback systems and emerging large language model (LLM) applications indicate potential decision-support roles, but they should be viewed as complementary tools rather than substitutes for expert judgment in the absence of robust, replicated validation [18,46,61,62].

From an applied perspective, the following implications should be read as practice-oriented interpretations derived from recurring patterns in the mapped evidence (i.e., descriptive and hypothesis-generating), rather than as prescriptive “best-practice” recommendations. The most consistently supported practical implications include: (1) prioritizing intensity-weighted external-load indicators (high-intensity jumps, accelerations/decelerations) alongside volume to improve day-to-day sensitivity to functional changes [29,36,38,50]; (2) triangulating external load with feasible internal-load/recovery measures (s-RPE, HRV, TQR/wellness) to contextualize the stress–recovery balance and reduce single-metric interpretations [13,40,41]; and (3) applying position- and modality-specific interpretation (indoor vs. beach; role demands) when setting monitoring targets and comparing athletes [23,24,46,60].

The future research priorities highlighted by this evidence map include: (i) longitudinal designs that integrate women-specific moderators and report sex-disaggregated analyses when mixed cohorts are used; (ii) standardized reporting of monitoring metrics and contextual variables to improve comparability; and (iii) transparent AI/ML pipelines with external validation, calibration, and interpretable outputs suitable for implementation in real-world settings.

### 4.2. Limitations

In this scoping review, several methodological considerations should be taken into account when interpreting the evidence presented. First, limiting the search to publications in English, Spanish, Portuguese, and Italian may have excluded relevant studies published in other languages, introducing potential language bias. This decision was primarily pragmatic, as it enabled a rigorous and consistent full-text assessment by the review team a common approach in evidence, mapping studies when specialized translation resources are limited.

Second, despite systematic retrieval efforts, a small number of potentially eligible studies could not be included due to restricted access to full texts. This may affect the comprehensiveness of the evidence map and may inadvertently lead to an overrepresentation of evidence from journals or regions with greater accessibility (e.g., open-access outlets or those covered by institutional subscriptions). Therefore, the findings should be understood as a synthesis of accessible and assessable evidence rather than as a fully exhaustive representation of the entire literature on the topic.

In addition, we did not perform critical appraisal of included sources; therefore, the review does not grade the strength of evidence and the findings should be interpreted as an evidence map rather than as effectiveness recommendations.

Finally, the methodological heterogeneity observed across included studies, regarding research designs, monitoring technologies, load metrics, analytical approaches, and outcome definitions, is inherent to the purpose and scope of a scoping review. Consequently, this variability precluded direct comparisons between studies and quantitative synthesis. Instead, the central contribution of this work is to describe recurring patterns, identify trends, and delineate priority research gaps, avoiding interpretation of the findings as conclusive evidence of superiority or comparative effectiveness of specific methods.

## 5. Conclusions

This scoping review mapped recent evidence on the use of non-invasive monitoring technologies and, less frequently, artificial intelligence/machine learning (AI/ML) approaches for training load management, fatigue monitoring, performance, and injury-related outcomes in women’s volleyball. Consistent with the purpose of a scoping review, the findings should be interpreted as a descriptive, hypothesis-generating synthesis of recurring patterns and methodological characteristics of the field, rather than as an assessment of comparative effectiveness.

Across the evidence map, monitoring-only studies predominated, particularly those using inertial measurement units (IMUs), force platforms, heart rate/heart rate variability (HR/HRV) monitoring, perceptual tools (e.g., s-RPE, TQR/wellness), and, depending on the modality, GPSs/LPSs. Consistently across designs and contexts, intensity-weighted external load indicators (e.g., exposure to high-intensity jumps and acceleration/deceleration demands) were more frequently linked to fatigue-sensitive signals than accumulated volume alone. Likewise, context (training vs. competition; indoor vs. beach; surface) and role/position emerged as relevant moderators of load interpretation, reinforcing the need for modality- and tactical-function-specific interpretive frameworks.

AI/ML applications were less frequent than instrumental monitoring and, when implemented, were mainly oriented toward multi-source integration and prediction/estimation of states such as fatigue/readiness or performance outcomes. However, the evidence map indicates recurring limitations that constrain practical transfer, especially regarding external validation, calibration, and interpretability.

A cross-cutting finding is that women-specific physiological moderators (e.g., menstrual-cycle-related variables or other biological modulators) are incorporated in a limited and unsystematic manner in study designs and reporting, which restricts the applicability of the evidence toward truly individualized monitoring in women’s volleyball. Accordingly, this map identifies priority gaps in standardized reporting (metrics and context), an increase in longitudinal designs with women-specific analyses, and the consolidation of transparent AI/ML pipelines with external validation and interpretable outputs.

From an applied perspective, and within the scope of an evidence-mapping review, the practical implications most consistently supported by the synthesized evidence are (1) prioritizing intensity metrics (high-intensity jumps, accelerations/decelerations) alongside volume to improve day-to-day sensitivity to functional changes; (2) triangulating multimodal monitoring (external load, internal/autonomic indicators, and perception) to contextualize the stress–recovery balance and reduce single-metric interpretations; and (3) interpreting results by context and role/position, avoiding global averages that may conceal meaningful within-team variability.

## Figures and Tables

**Table 1:** PCC Methodology.

Population (P)	Female volleyball athletes participating at any organized competitive level, including competitive amateur, scholastic, regional, collegiate, national, semi-professional, and professional categories.
Concept (C)	Non-invasive monitoring technologies to quantify external and internal load, neuromuscular/perceptual fatigue, and/or performance readiness, and artificial intelligence/machine learning (AI/ML) approaches used for multi-source integration, classification, or prediction (e.g., fatigue/readiness). AI/ML will be considered only when models are trained with an explicit validation procedure and report performance metrics, distinguishing them from conventional statistical analyses.
Context (C)	Training and competition environments, including laboratory-based studies that evaluate technologies or algorithms applicable to game contexts, considering sport-specific execution actions such as explosive tasks, multiple directional changes, and essential technical–tactical components of the discipline.

## Data Availability

No new data were created or analyzed in this study. All data extracted and synthesized derive from previously published articles included in the scoping review. Data sharing is therefore not applicable.

## Supplementary Materials

The following supporting information can be downloaded at: https://www.mdpi.com/article/10.3390/sports14020074/s1, File S1: PRISMA-ScR Checklist; Table S1: Complete Search Strategy for Scopus, Web of Science, and PubMed.; Table S2. Extraction Matrix of Included Studies. Reference [63] is cited in the supplementary materials

## Abbreviations

The following abbreviations are used in this manuscript:

ACWR	Acute:Chronic Workload Ratio
AI	Artificial Intelligence
CMJ	Countermovement Jump
EWMA	Exponentially Weighted Moving Average
HR	Heart Rate
GPS	Global Positioning System
HRV	Heart Rate Variability
ICC	Intraclass Correlation Coefficient
IMU	Inertial Measurement Unit
LLM	Large Language Model
LPS	Local Positioning System
MeSH	Medical Subject Heading
PPG	Photoplethysmography
rMSSD	Root Mean Square of Successive Differences
RPE	Rating of Perceived Exertion
Session-RPE (s-RPE)	Session Rating of Perceived Exertion
STR	Stress Score (Workload Metric)
UWB	Ultra-Wideband
VO_2_max	Maximal Oxygen Uptake
