# Supplementary material for: Machine Learning and Non-Invasive Monitoring Technologies for Training Load Management in Women’s Volleyball: A Scoping Review
Source: Sports (Basel). 2026 Feb 7;14(2):74. doi: 10.3390/sports14020074 (PMC12944405; doi:10.3390/sports14020074)
Supplement: Supplementary file 1 [file sports-14-00074-s001.zip › Supplementary_Table_S1_Search_Strategy_MDPI_Sports_EN.pdf]

Supplementary Table S1. Complete search strategy for Scopus, Web of Science, and PubMed.

Search coverage: January 2020 to September 2025; no systematic search of grey literature or preprints. Records identified: Scopus (n = 148), Web of Science (n = 145), PubMed (n = 46).

**Database**	**Search fields / syntax**	**Complete search string (verbatim)**	**Limits / filters applied**	Records identified (n)
Scopus	TITLE-ABS-KEY	TITLE-ABS-KEY (volleyball OR "beach volleyball" OR voleibol OR volei OR pallavolo) AND (TITLE-ABS-KEY ("artificial intelligence" OR "machine learning" OR "deep learning" OR "neural network" OR "neural networks" OR "random forest" OR "random forests" OR "support vector machine" OR "support vector machines" OR SVM OR XGBoost OR "gradient boosting" OR algorithm*) OR TITLE-ABS-KEY (wearable* OR sensor* OR acceleromet* OR "inertial measurement unit" OR "inertial measurement units" OR IMU OR gyroscop* OR magnetomet* OR actigraph* OR "force plate" OR "force plates" OR PPG OR photoplethysmograph* OR "heart rate variability" OR HRV OR "heart rate monitor" OR "heart rate monitors" OR "skin temperature" OR thermograph* OR "local positioning system" OR "ultra wideband" OR "ultra-wideband" OR UWB OR GPS OR "session RPE" OR "session-RPE" OR s-RPE OR "rate of perceived exertion" OR RPE)) AND TITLE-ABS-KEY ("training load" OR workload* OR "external load" OR "internal load" OR fatigue OR readiness OR recovery OR neuromuscular OR "jump performance" OR "jumping performance" OR CMJ OR "countermovement jump") AND PUBYEAR > 2019 AND PUBYEAR < 2026 AND (LIMIT-TO (DOCTYPE , "ar") OR LIMIT-TO (DOCTYPE , "re") OR LIMIT-TO (DOCTYPE , "cp")) AND (LIMIT-TO (LANGUAGE , "English") OR LIMIT-TO (LANGUAGE , "Spanish") OR LIMIT-TO (LANGUAGE , "Portuguese") OR LIMIT-TO (LANGUAGE , "Italian"))	Coverage: 2020–2025 (PUBYEAR > 2019 AND PUBYEAR < 2026)<br>Doc types: "ar" OR "re" OR "cp"<br>Languages: English, Spanish, Portuguese, Italian	148
PubMed	MeSH + Title/Abstract	(volleyball[MeSH Terms] OR volleyball[Title/Abstract] OR "beach volleyball"[Title/Abstract] OR voleibol[Title/Abstract] OR "pallavolo"[Title/Abstract] OR "volei"[Title/Abstract]) AND (("Artificial Intelligence"[MeSH] OR "Machine Learning"[MeSH] OR "deep learning"[Title/Abstract] OR "neural network"[Title/Abstract] OR "neural networks"[Title/Abstract] OR "machine learning"[Title/Abstract] OR "artificial	Coverage: "2020/01/01"–"2025/09/30" (Date - Publication)<br>Species: Humans<br>Languages: English, Spanish, Portuguese, Italian<br>Publication types: Journal Article, Review, Clinical	46

		intelligence"[Title/Abstract] OR "random forest"[Title/Abstract] OR "random forests"[Title/Abstract] OR "support vector"[Title/Abstract] OR "support vector machine"[Title/Abstract] OR "support vector machines"[Title/Abstract] OR SVM[Title/Abstract] OR XGBoost[Title/Abstract] OR algorithm[Title/Abstract] OR algorithms[Title/Abstract]) OR ("Wearable Electronic Devices"[MeSH] OR wearable[Title/Abstract] OR wearables[Title/Abstract] OR sensor[Title/Abstract] OR sensors[Title/Abstract] OR accelerometer[Title/Abstract] OR accelerometers[Title/Abstract] OR "inertial measurement unit"[Title/Abstract] OR "inertial measurement units"[Title/Abstract] OR IMU[Title/Abstract] OR gyroscope[Title/Abstract] OR gyroscopes[Title/Abstract] OR actigraph[Title/Abstract] OR actigraphy[Title/Abstract] OR "force plate"[Title/Abstract] OR "force plates"[Title/Abstract] OR photoplethysmography[Title/Abstract] OR photoplethysmograph[Title/Abstract] OR PPG[Title/Abstract] OR "heart rate variability"[Title/Abstract] OR HRV[Title/Abstract] OR "heart rate monitor"[Title/Abstract] OR "heart rate monitoring"[Title/Abstract] OR thermography[Title/Abstract] OR thermograph[Title/Abstract] OR "skin temperature"[Title/Abstract] OR "local positioning"[Title/Abstract] OR "ultra-wideband"[Title/Abstract] OR UWB[Title/Abstract] OR GPS[Title/Abstract] OR "session-RPE"[Title/Abstract] OR "session RPE"[Title/Abstract] OR s-RPE[Title/Abstract] OR "rate of perceived exertion"[Title/Abstract] OR RPE[Title/Abstract])) AND ("training load"[Title/Abstract] OR workload[Title/Abstract] OR workloads[Title/Abstract] OR "external load"[Title/Abstract] OR "internal load"[Title/Abstract] OR fatigue[Title/Abstract] OR readiness[Title/Abstract] OR recovery[Title/Abstract] OR neuromuscular[Title/Abstract] OR "jump performance"[Title/Abstract] OR "jumping performance"[Title/Abstract] OR CMJ[Title/Abstract] OR "countermovement jump"[Title/Abstract]) AND ("2020/01/01"[Date - Publication] : "2025/09/30"[Date - Publication]) AND (humans[MeSH Terms]) AND (English[Language] OR Spanish[Language] OR Portuguese[Language] OR	Trial, Randomized Controlled Trial, Validation Study, Observational Study	

		Italian[Language]) AND ("Journal Article"[Publication Type] OR "Review"[Publication Type] OR "Clinical Trial"[Publication Type] OR "Randomized Controlled Trial"[Publication Type] OR "Validation Study"[Publication Type] OR "Observational Study"[Publication Type])		
Web of Science	TS (Topic)	TS=(volleyball OR "beach volleyball" OR voleibol OR vôlei OR pallavolo) AND (TS=("artificial intelligence" OR "machine learning" OR "deep learning" OR "neural network*" OR "random forest*" OR "support vector*" OR SVM OR XGBoost OR "gradient boosting" OR algorithm*) OR TS=(wearable* OR sensor* OR acceleromet* OR "inertial measurement unit*" OR IMU OR gyroscop* OR magnetomet* OR actigraph* OR "force plate*" OR PPG OR photoplethysmograph* OR "heart rate variab*" OR HRV OR "heart rate monitor*" OR "skin temperature" OR thermograph* OR "local positioning system*" OR "ultra-wideband" OR UWB OR GPS OR "session-RPE" OR s-RPE OR "rate of perceived exertion" OR RPE)) AND TS=("training load" OR workload* OR "external load" OR "internal load" OR fatigue OR readiness OR recovery OR neuromuscular OR "jump* performance" OR CMJ OR "countermovement jump") AND PY=(2020-2025) AND LA=(English OR Spanish OR Portuguese OR Italian) AND DT=(Article OR Review OR "Proceedings Paper" OR "Early Access")	Coverage: 2020–2025 (PY)<br>Languages: English, Spanish, Portuguese, Italian (LA)<br>Document types: Article, Review, Proceedings Paper, Early Access (DT)	145
