# Supplementary material for: Machine Learning and Non-Invasive Monitoring Technologies for Training Load Management in Women’s Volleyball: A Scoping Review
Source: Sports (Basel). 2026 Feb 7;14(2):74. doi: 10.3390/sports14020074 (PMC12944405; doi:10.3390/sports14020074)
Supplement: Supplementary file 1 [file sports-14-00074-s001.zip › Supplementary_Table_S2_Extraction_Matrix.pdf]

Supplementary Table S2. Extraction Matrix of Included Studies

No.	Authors (Year)	Article title	Country	Primary aim	Sample	Age	Technology/AI	Load monitoring domain	Key findings
1	Pajo LS, Rabuya R, Andacao A, Tuano AMS, Lobo J (2025) [62]	A 10-week LLM-generated versus human-made volleyball training program on the jumping performance of collegiate volleyball athletes	Philippines	compare the effectiveness of A program of training generated by AI (ChatGPT 3.5) versus uno diseñado by humans to improve the performance of jump vertical and horizontal in athletes collegiate of volleyball	43 athletes collegiate of volleyball; Group AI: *n* = 23 (12 women, 11 men); Group human: *n* = 20 (10 women, 10 men)	athletes collegiate (Age específica no reportada)	LLM ChatGPT 3.5 to generación of program of training of 10 weeks, 3 sessions/week	Focus in optimization of the performance through prescripción algorithmic; assessment pre-post of capacityes of jump	Programa AI: improvements in jump vertical unilateral derecho and horizontal unilateral derecho; efectos significativos in women to jump vertical bilateral. Programa human: superior in jump vertical bilateral, especialmente in women. Conclusion: AI tiene potencial pero aún no sustituye completamente programs profesionales
2	Makaracı Y, Makaracı M, Zorba E, Lautenbach F (2023) [46]	A Pilot Study of the Biofeedback Training to Reduce Salivary Cortisol Level and Improve Mental Health in	Turkey (colaboración with Germany)	examine the efectos of 7 weeks of training with biofeedback HRV on respuestas acute and chronic of stress endocrino, actividad	6 volleyball players female highly trained (competition nacional)	17.50 ± 1.05 years (range 17-19 years)	Sistema Nexus 10 with software Bio Trace to biofeedback HRV; monitoreo no invasivo of cortisol salival, HRV (RMSSD, SDNN), heart rate; analizador automatizado Unicel 800 DxI to cortisol	assessment multidimensional of fatigue: cortisol salival (CAR and medio día) as biomarker of stress, HRV as indicador of function autonomic, DASS-21 to mental health; aplicación to readiness and	reduction significant of the cortisol of medio día post-intervención (Z=2.201, p=0.028; -26.58% mean). reduction significant of the cortisol intra-session in 6/8 sessions evaluadas (-22.16% mean). improvements significativas in mental health (DASS-21: anxiety, stress, score total).

No.	Authors (Year)	Article title	Country	Primary aim	Sample	Age	Technology/AI	Load monitoring domain	Key findings
		Highly-Trained Female athletes		parasimpática and mental health in athletes female of volleyball highly trained				management of internal load	Tendencias no significativas of improvement in HRV. Limitations: n small (*n* = 6), design without group control
3	Bonnette S, DiCesare CA, Kiefer AW, Riley MA, Barber Foss KD, Thomas S, Diekfuss JA, Myer GD (2020) [61]	A Technical Report on the Development of A Real-Time Visual Biofeedback System to Optimize Motor Learning and Movement Deficit Correction	United States	Develop e implementar A sistema of biofeedback visual in real time que integre múltiples variables biomechanical of risk of injury of the LCA in A sola pantalla interactiva to optimizar the aprendizaje motor and reducir factors of risk	11 athletes female adolescent of volleyball varsity	16.7 ± 1.34 years	Sistema integrado: capture of motion 10 cameras Raptor-E (240 Hz), platforms of force BP600900 AMTI (1200 Hz), algorithms personalizados C++ with OpenGL, HMD wireless to biofeedback visual, analysis heat map algorithmic; proceswithoutg in real time of 4 variables biomechanical simultaneous	Optimización biomechanical through monitoreo integrado of: inclinación of the trunk, ratio moment extensor knee-hip (KHMr), moment abducción knee (KAM), symmetry force of reacción vertical (vGRF); aplicación to injury prevention and corrección of déficits neuromusculares	improvement sig. in heat map scores: 77.17% → 84.87% (+7.70%, t=5.16, p<0.001, d=1.56, power=0.99). Transferencia exitosa to the DVJ: greater flexión knee (-2.63°, p=0.02, d=0.86), greater flexión hip (+3.16°, p=0.047, d=0.68), lower moment extensor knee (+10.25 Nm, p=0.002, d=1.28). Demostración of feasibility técnica to integración of data biomecánicos in real time
4	Sanders GJ, Skodinski S, Peacoc	Analyzing the Impact of Various Jump	United States	Crear A focus systematic to analyze data to	9 athletes of women's volleyball División I NCAA	19.4 ± 1.3 years; height: 184.0 ± 7.1 cm (peso	VERT 3 IMU validado (accelerometer 3D, gyroscope, magnetometer) to monitoreo loads jump	Monitoreo longitudinal integrado external load-interna: loads	Correlaciones more significativas: Jumps 50+ with métricas CMJ mean mostraron correlaciones negativas more fuertes

No.	Authors (Year)	Article title	Country	Primary aim	Sample	Age	Technology/AI	Load monitoring domain	Key findings
	k CA (2025) [33]	Load Intensities on Countermovement Jump Metrics: A Comparison of Average, Peak, and Peak-to-Average Ratios in Force-Based Metrics		improve models predictive of fatigue and performance neuromuscular in volleyball, determining which métricas CMJ (mean, pico, ratios pico-mean) predict more precisamente changes neuromusculares after loads of jump daily		excluido by restricciones institucionales)	continuo; Hawkin Dynamics platform dual force portable (1000 Hz); models regression linear predictive; analysis statistical SPSS with validation power analysis (0.80); framework to future algorithms ML	jump daily categorizadas (total, >38.1cm [Jumps 38+], >50.8cm [Jumps 50+]) correlacionadas with métricas CMJ día siguiente (height, tiempo vuelo, RFD frenado, profundidad contramotion, forces frenado/propulsión, RSI, mRSI); assessment predictiva neuromuscular fatigue; optimization distribución loads basada in respuestas individuales	(r=-0.384 A -0.529, p<0.001 to todas). Capacidad predictiva: Métricas mean CMJ providesron mejor capacity predictiva que métricas pico (R^2 up to 0.280, explicando up to 28% variabilidad in neuromuscular fatigue). Ratios pico-mean: without relaciones significativas with loads (p≥0)
5	Marković S, Dopsaj M, Tomažič S, Kos A, Nedeljković A, Umek A	Can IMU Provide an Accurate Vertical Jump Height Estimate?	Eslovenia/ Serbia	determine si A inertial measurement unit colocada in the parte metatarsal of the pie puede providesr data válidos	13 volleyball players female of elite (team nacional of Serbia)	24.6 ± 3.2 years; height: 187.8 ± 4.3 cm; peso: 75.0 ± 3.87 kg; experience: 13.5 ± 3.5 years	IMU personalizado: LSM6DS33 accelerometer/gyroscope 3D (±16 g_0, 200 Hz), microcontrolador Adafruit Feather M0 WiFi, transmisión UDP inalámbrica, LabView 2019; algorithms: filtro Butterworth (orden=5, fcof=40 Hz), método	Cuantificación precisa capacityes explosivas through height jump vertical as indicador estado neuromuscular and fatigue; assessment modalities CMJ	Validez concurrente excelente: CMJ: ICC=0.975, t=0.897, p=0.379, d=0.176 (negligible); bias=-0.18 cm, LOA=-2.26 A 1.9 cm, McV=1.896%. SQJ: ICC=0.921, t=-0.564, p=0.578, d=0.111 (negligible); bias=0.18 cm, LOA=-3.14 A 3.5 cm,

No.	Authors (Year)	Article title	Country	Primary aim	Sample	Age	Technology/AI	Load monitoring domain	Key findings
	(2021) [31]			and confiables to estimación precisa of height of jump vertical in modalities CMJ and SQJ in volleyball players of elite			tiempo of vuelo, detección automática eventos (threshold 5 g_0), validation vs. platform force AMTI (1000 Hz)	(ciclo estiramiento-acortamiento) and SQJ (fase concéntrica aislada); sistema portable to monitoreo continuo readiness without limitations laboratorio; aplicación to seguimiento estado training and ajustes of load	McV=3.556%. Confiabilidad alta: CMJ: ICC=0.888, McV=4.116%; SQJ: ICC=0.872, McV=5.933%. Alturas mean: CMJ_FP=30.29±3.41 cm vs CMJ_IMU=30.11±3.25 cm; SQJ_FP=27.36±2.67 cm vs SQJ_IMU=27.54±3
6	Schumann C, Wojciechowski M, Bunn JA (2023) [50]	Comparing Two Methods of Acute: Chronic Workload Calculations in Girls' Youth Volleyball	United States (Texas)	compare changes weekles in producción of energía cinética in athletes youthes female during seasons of secundaria and club, and evaluate concordancia between métodos of cálculo ACWR	24 athletes of women's volleyball youth: 12 HSVB (varsity 6A) + 12 CVB (club elite Texas)	athletes of secundaria (14-18 years, Age específica no reportada)	Validated VERT model KMT (accelerometer/gyroscope/magnetometer), iOS wireless transmission, myVERT cloud storage; ACWR computed uwithoutg Rolling Average vs EWMA.	Monitoreo longitudinal integrado usando energía cinética (J/lb) as external load; implementación ACWR to mantener range óptimo (0.8-1.5): fitness maximal + risk injury mínimo; detección automática picos load (>1.5) and valles destraining (<0.8); comparación	HSVB (season consistente): ACWR mantuvo range óptimo greatería weeks, picos significativos inicio season and week 7-8. correlation fuerte RA-EWMA (r=0.756, p<0.001). EWMA more sensible detectando picos (mejor prevención injuries). CVB (season inconsistente):Mayor variabilidad weekly (p<0.05), múltiples weeks outside range óptimo, valles significativos weeks 4,13,19. correlation

No.	Authors (Year)	Article title	Country	Primary aim	Sample	Age	Technology/AI	Load monitoring domain	Key findings
				(Rolling Average vs. Exponentially Weighted Moving Average)				metodológica seasons consistentes (HSVB: 6 días/week, 4 meses) vs. inconsistentes (CVB: 2 trainings/week + torneos intensivos, 7 meses)	moderada RA-EWMA (r=0)
7	Foster E, Bunn JA (2024) [25]	Comparison of External Load across Multi-Day Tournaments in Female Youth Volleyball athletes	United States	evaluate differences in external load of athletes youthes female of volleyball A través of días and by posición during torneos multi-día, evaluando efectos acumulativos of fatigue and recovery	22 athletes of women's volleyball youth of club (OH: *n* = 8, MB: *n* = 6, SET: *n* = 4, DS/L: *n* = 4)	16.4 ± 0.4 years	Validated VERT model KMT (triaxial accelerometer and gyroscope) with VERT Team System iOS app; outputs included kinetic energy (KE, J/lb), total jumps, high jumps (>51 cm), mean jump height, and movement distribution.	Monitoreo external load during torneos multi-día (2-3 días consecutivos, 3-4 matches/día): assessment fatigue acumulativa and recovery nocturna; analysis diferencial positional to optimizar estrategias recovery; detección tolerancia competitiva in athletes youthes; aplicación to prevención sobretraining in	without differences between días: Todas the variables external load mantuvieron valores similar días 1-3 (KE: 484.8-558.0 J/lb, TJ: 27.3-31.2/partido, height: 37.5-37.9 cm, HJ: 5.2-6.5/partido, stress: 20.1-20.7%, p≥0.143), indicando recovery nocturna suficiente. difference positionales significativas: SET greater TJ que DS/L (p=0.005); MB greater height que todas positions (p=0.000-0.044)

No.	Authors (Year)	Article title	Country	Primary aim	Sample	Age	Technology/AI	Load monitoring domain	Key findings
								competition alta densidad	
8	Kipp K, Kiely M, Geiser C (2021) [34]	Competition Volume and Changes in Countermovement Jump Biomechanics and Motor Signatures in Female Collegiate Volleyball players	United States	investigate the relationship between volumen competitivo and changes pre-post season in biomechanical CMJ and firmas motoras in players universitarias of volleyball, evaluando efectos specific by posición and volumen individual of competition	10 volleyball players female División I NCAA (3 defensive specialists, 5 hitters, 2 setters)	20 ± 1 years; height: 1.81 ± 0.10 m; peso: 71.6 ± 7.6 kg	Plataformas of force duales AMTI (1000 Hz), filtro Butterworth 15 Hz, proceswithoutg MATLAB personalizado; analysis factorial: componentes principales with rotación VARIMAX (Kaiser-Meyer-Olkin=0.649, Bartlett p<0.001), reduction dimensional 4→2 factors, factor scores método regression; variables biomechanical: PeakF, PeakRFD, TIME, EccT:TIME; confiabilidad ICC 0.669-0.983; analysis statistical SPSS 22	Caracterización integral respuestas load competitiva: correlation volumen competitivo (sets jugados: 94.5±29.7 during 95 días, 33 matches) with changes biomecánicos CMJ pre-post season; identificación firmas motoras individualizadas (factors "force" and "velocity") to optimization específica by posición; detección adaptaciones neuromusculares longitudinales	correlation load-respuesta sig.: Mayor volumen competitivo correlacionó negativamente with changes height CMJ (r=-0.715, p=0.020). Cambios biomecánicos post-season: increase sig. TIME (+0.065±0.075s, p=0.026, ES=0.57) and PeakRFD (+27.7±38.8 $N\cdot kg^{-1}\cdot s^{-1}$, p=0.040, ES=0.55). Firmas motoras estables: Factor 1 "force" (PeakF, PeakRFD, 56.1% varianza) and Factor 2 "velocity" (TIME, EccT:TIME, 25
9	Jimenez-Olmedo JM, Pueo B, Mossi JM, Villalon	Concurrent Validity of the Inertial Measurement Unit Vmaxpro	Spain	quantify the validez concurrente of the IMU Vmaxpro to estimar height of jump	13 volleyball players female highly trained (Liga Superliga 2	22.23 ± 3.26 years; height: 1.72 ± 0.06 m; peso: 64.12 ± 7.33 kg; experience: 8.77 ± 2.01	IMU Vmaxpro (accelerometer/gyroscope/magnetometer triaxiales, 1000 Hz, transmisión Bluetooth 65 Hz, app iOS) colocada in hip; MoCAP Optitrack (6 cameras Flex 3, 100	validation of herramienta portable to cuantificación precisa of VJH as indicador clave estado neuromuscular	correlation fuerte: Spearman rs=0.844 (p<0.001, muy alta). Concordancia débil: CCC=0.219 (ρ=0.861 accuracy alta, Cb=0.261 exactitud baja). Error systematic sig.: Vmaxpro

No.	Authors (Year)	Article title	Country	Primary aim	Sample	Age	Technology/AI	Load monitoring domain	Key findings
	-Gasch L (2023) [30]	in Vertical Jump Estimation		vertical (VJH) in CMJ comparado with sistema of capture of motion 3D (MoCAP) as gold standard in volleyball players female highly trained	española)	years	Hz, marcador L4-L5); algorithms Vmaxpro to estimación VJH from velocity of despegue (VJH = $v_0^2/2g$); analysis statistical MedCalc: Wilcoxon, Spearman, CCC Lin, regression Paswithoutg-Bablok, Bland-Altman	and fatigue; permite monitoreo readiness of the atleta and respuesta A loads training; facilita toma decisiones management of load through alternativa económica A sistemas laboratorio; aplicación ecuación ajuste (HMoCAP = 8.463 + 1.067 HVmaxpro) to corrección error systematic	subestima 10.52 cm vs MoCAP (p<0.001, ES=2.39). Regresión Paswithoutg-Bablok:HMoCAP = 8.463 + 1.067 HVmaxpro (intercepto 8.46 cm, pendiente 1.067≈1), SEE=1.67 cm (error aleatorio moderado). Bland-Altman:sesgo systematic 10.6 cm, LoA 5.96-15.2 cm
10	Miranda-Mendoza J, Hernández-Cruz G, Reynoso-Sánchez LF, González-Fimbres RA, Cejas	Control of recovery uwithoutg the Total Quality Recovery (TQR) scale during four accumulation microcycles and its relationsh	Mexico	Analyze the behavior of the modified TQR scale across four accumulation microcycles and its relationships with physiological markers (HRV, TRIMP), perceived	7 volleyball players female (selección nacional)	24.26 ± 3.37 years	Polar Team 2 (HRV-RR intervals), TRIMP (Edwards), TQR (escala 0-10), sRPE (Borg), RESTQ-Sport; proceswithoutg Kubios software; without AI/ML	Daily multidimensional monitoring of internal load and recovery for 21 days; assessment of TQR sensitivity to cumulative microcycles; correlations between physiological and perceptual markers.	TQR mostró correlaciones significativas with LnrMSSD (r=0.69, p<0.05) and Stress Score (r=-0.64, p<0.05); correlaciones inversas with sRPE and dimensiones of stress of RESTQ-Sport; LnrMSSD estable in M1-M3 with descenso significant in M4; TQR mantuvo valores of recovery insuficiente (<7) during the cuatro microciclos;

No.	Authors (Year)	Article title	Country	Primary aim	Sample	Age	Technology/AI	Load monitoring domain	Key findings
	Hernández BA (2023) [41]	ip to physiological factors		load (sRPE), and recovery-stress status (RESTQ-Sport).					validation concurrente of TQR as herramienta práctica to monitoreo of recovery
11	Hank M, Cabell L, Zahalka F, Miřátský P, Cabrnoch B, Mala L, Maly T (2024) [37]	Differences in external load among indoor and beach volleyball players during elite matches	República Checa, United States	examine differences in parameters of external load between volleyball playa e indoor and between positions individuales during tiempo activo of juego in matches of elite females	22 players of elite (8 playa: BlockerBV *n* = 4, DefenderBV *n* = 4; 14 indoor: Blocker *n* = 4, Setter *n* = 2, Libero *n* = 2, Outside *n* = 4, Opposite *n* = 2)	28.6 ± 5.8 years; Indoor: 25.2 ± 6.1 years	Videografía multicámara HD (Sony HDC90E, 50 fps), TEMA Trackeye v2.3 to analysis cinemático 3D and seguimiento semi-automático, MATLAB to cálculo of métricas (DR, TD, rTD, PL, rPL, ePL); fórmula cartesiana of Nicolella et to the. to Player Load™; without AI/ML	Cuantificación of external load by rally individual (2,336 trayectorias): duración, distancia total and relativa, Player Load™ estándar/relativo/explosivo; analysis diferencial by modalidad and posición to optimization of training específico	Playa vs Indoor: greater valores TD (+6.6%), rTD (+10.8%), PL (+10.5%), rPL and ePL (up to +23%) in playa (p<0.05, efectos small-medianos). 80% rallies ≤10s both modalities; 62% rallies with 4.5-10m distances covered. positions: Libero reached loads similar A players playa; Blocker indoor + Libero > Setter + Outside + Opposite in several parameters. Playa: without differences BlockerBV vs DefenderBV
12	Andrade AD, Simim M, Kassiano W, Palao JM, de Jesus K, Maia N, Assumpção C,	Do differences between the training load perceived by elite beach volleyball players and that	Brazil, United States	Verificar differences between load of training planificada by coaches vs. percibida by volleyball players playa of elite and	3 volleyball players playa female of elite (top 10 ranking nacional, 2 clasificadas Juegos Olímpicos 2021)	23.2 ± 2.2 years; 1.80 ± 0.1 m; 79.0 ± 6.0 kg; 16.5 ± 1.6% grasa corporal	Plataforma of contacto Chronojump to CMJ (6 intentos mean, 60s descanso between repeticiones), escala RPE Borg (0-10), cálculo sRPE (duración × RPE); analysis statistical through differences estandarizadas medias (SMD) e inferencia clínica; without AI/ML	Monitoreo diario during 3 weeks preseason: RPE coaches vs. athletes diferenciado by tipo of training (force, acondicionamiento físico, tactical-technical), internal load	week 1: differences substantial RPE coaches vs. athletes (SMD=-1.29, IC90%=-1.99;-0.58, 99/01/00). training force and acondicionamiento: differences likely (force: SMD=0.43, 80/19/02; acondicionamiento: SMD=0.40, 78/21/01). training tactical-technical: without

No.	Authors (Year)	Article title	Country	Primary aim	Sample	Age	Technology/AI	Load monitoring domain	Key findings
	Medeiros AIA (2020) [22]	planned by coaches affect neuromuscular function?		observar efectos on function neuromuscular during preseason				total (sRPE), assessment weekly function neuromuscular (CMJ lunes pre-training); analysis of concordancia load planificada-percibida	differences significativas. CMJ: increase sustancial week 1→3 (SMD=1.60, IC90%=0.00;3.21, 93/03/04, +7% improvement)
13	Edmonds R, Schmidt B, Siedlik J (2021) [44]	Eligibility Classification as A Factor in Understanding Student-Athlete Responses to Collegiate Volleyball Competition	United States	examine differences in HRV between estudiantes-athletes of diferentes clasificaciones of elegibilidad (freshman, sophomore, junior, senior) and analyze differences in HRV to the competir in casa vs. visitante during season universitaria completa	14 volleyball players female División I NCAA (Freshmen *n* = 3, Sophomores *n* = 3, Juniors *n* = 4, Seniors *n* = 4)	Total: 20.4 ± 1.5 years; Freshmen: 18.0 ± 0.0; Sophomores: 20.0 ± 0.0; Juniors: 21.0 ± 0.8; Seniors: 21.8 ± 0.5 years	Sensor infrarrojo ithlete (HRV Fit Ltd.) validado, aplicación smartphone to HRV (lnRMSSD×20, respiración controlada 7.5 resp/min, eliminación automática artefactos), VAS 1-9 to autoreporte wellness (sleep, fatigue, dolor muscular, stress, estado of ánimo); analysis statistical JASP with ANOVA and correlaciones Spearman; without AI/ML	Monitoreo diario matutino HRV during season completa (30 matches): comparaciones pre-gameday/gameday/post-gameday, casa vs. visitante, analysis diferencial by classification elegibilidad académica; assessment multidimensional fatigue through biomarker aim (HRV) + measures subjetivas wellness integradas	difference by classification: Freshmen HRV significativamente lower (80.3±9.7) vs. Sophomores (85.7±7.2), Juniors (91.2±8.3), Seniors (86.5±7.2) ($p<0.05$, $\eta^2=0.17$); Freshmen peor estado ánimo (6.0±1.1) vs. otras clasificaciones ($p<0.05$, $\eta^2=0.23$); Juniors HRV more alta vs. Sophomores/Seniors; Junior/Senior greater stress autorreportado vs. Freshman/Sophomore. Casa vs. Visitante
14	Bozzini BN,	Evaluation of	United States	evaluate loads of	20 players volleyball	20 ± 1 years (range 18-	Polar TeamPro system (GPS, heart rate,	Monitoreo longitudinal 6	Características performance: Travel

No.	Authors (Year)	Article title	Country	Primary aim	Sample	Age	Technology/AI	Load monitoring domain	Key findings
	McFadden BA, Scruggs SK, Arent SM (2021) [28]	Performance Characteristics and Internal and External Training Loads in Female Collegiate Beach Volleyball players		training internas and externas during season competitiva completa and quantify características of performance in players universitarias División I NCAA of volleyball playa female, incluyendo analysis diferencial between travel and non-travel squads	playa female División I NCAA (travel squad *n* = 11, non-travel squad *n* = 7, 2 alternantes excluidas of the subanalysis; program top 20 nacional)	23); height: 175.3 ± 5.2 cm; peso: 68.3 ± 6.3 kg; 21.80 ± 3.5% grasa corporal	accelerometry) individualized by age, height, body mass, VO_2max, HRVT and HRmax (from prior testing); Just Jump Mat for CMJ/CMJHOH (3 trials, 30 s rest); Vertec and TENDO for jump/velocity metrics.	weeks (3 preseason + 3 competitiva, truncada COVID-19): 13 sessions force/acondicionamiento (57 min mean), 34 prácticas (121 min mean), 12 duales competitivos (46 min mean); métricas TL, EEE absoluto/relativo (kcal and kcal/kg), distancia total, zonas HR/velocity individualizadas, accelerations/desaceleraciones categorizadas (4 zonas cada A), sprints (>2.8 m/s^2)	squad significativamente superior A non-travel in jump vertical (withoutgle-arm reach: p=0.028, ES=1.20; volleyball approach: p=0.024, ES=1.31) and velocity jump maximal (p=0.049, ES=1.05); non-travel greater VT (p=0.022, ES=-1.23); without differences composición corporal. Dinámica loads temporales: Interacciones tiempo×group significativas (p<0.001) to TL, EEE, distancia, HRZ1-5
15	Pawlik D, Mroczek D (2022) [29]	Fatigue and Training Load Factors in Volleyball	Poland	determine relationship between indicadores of external load (ETL) e interna (ITL) in volleyballist as female	11 players women's volleyball U19 (of 14 iniciales, 3 excluidas by participación incompleta	16-18 years; height: 172.42 ± 7.3 cm; peso: 63.54 ± 8.36 kg; IMC: 21.53 ± 2.93	Catapult Vector S7 with ClearSky (10 Hz positioning; 3D accelerometer ±16G; gyroscope; magnetometer) with individual calibration; Optojump for jump tests (SJ, CMJ, CMJA, etc.); SmartSpeed electronic	Monitoreo 5 días microciclo preparatory playoff nacional: measurement diaria ETL (Player Load, jumps totales by categorías, accelerations/des	Total accelerations were A better predictor of fatigue than total jumps; significant correlations with RPE (r = 0.480, p < 0.01) and sRPE (r = 0.515, p < 0.01), whereas total jumps were not significant; predictive models supported

No.	Authors (Year)	Article title	Country	Primary aim	Sample	Age	Technology/AI	Load monitoring domain	Key findings
				during microciclo weekly in season competitiva, identificando predictors of fatigue through analysis of accelerations, jumps and percepción subjetiva of the esfuerzo	)		timing gates for sprint tests.	aceleraciones categorizadas, heart rate mean) e ITL (TQR pre-training, RPE/sRPE post-training 30 min después); testing pre-post microciclo (jumps SJ/CMJ/CMJA/AJ/BJ, sprint 20m); analysis correlacional ETL-ITL and models predictive fatigue	acceleration-based indicators.
16	João PV, Medeiros A, Ortigão H, Lee M, Mota MP (2021) [36]	Global Position Analysis during Official Elite Female Beach Volleyball Competition: A Pilot Study	Portugal, Brazil, United Kingdom	quantify through technology GPS the demandas físicas of competition of volleyball playa female with referencia A posición of the player (Blocker vs. Defender), set and resultado of the partido to optimizar	12 players profesionales volleyball playa (6 Defenders, 6 Blockers) of top 6 teams Campeonato Nacional Portugués (of 38 teams totales)	Defenders: 26.3 ± 5.8 years, 175.3 ± 5.5 cm, 64.6 ± 4.0 kg, 9.6 ± 4.9 years experience; Blockers: 29.0 ± 7.7 years, 178.0 ± 5.2 cm, 66.1 ± 7.7 kg, 12.1 ± 6.1 years experience (10.9 ± 5.5 years mean experience	GPS 10 Hz with unidad measurement inercial (Minimax S4, Catapult Sports) in arnés C7-T2, activación 10 min pre-partido to desload efemérides satelitales, monitoreo real time exclusivo during tiempo activo; variables: distancia total/relativa, velocidades categorizadas (Low 0-3.9, Medium 4.0-6.9, High 7.0-12	Monitoreo 30 matches oficiales (50 sets: 16 primeros/segundos, 6 terceros, 8 byes) during torneo campeonato nacional; analysis external load diferenciada by posición (Blocker vs. Defender), variaciones intra-partido by set, identificación factors asociados with victoria/derrota	difference positionales significativas: Defenders > Blockers in Peak Player Load (6.6±3.1 vs 5.9±3.3 AU, p<0.001, ES=-0.20), distancia jogging (13.1±18.7% vs 4.4±3.8%, p<0.001, ES=-0.59), quick running (1.4±3.1% vs 0.1±0.5%, p<0.001, ES=-0.54), desaceleración medium (27.7±12.1% vs 25.2±9.8%, p<0.001) and high (14.2±13.3% vs 12.2±11.3%, p=0.041), jumps medium (53.2±22.1% vs 47.7±22.0%, p<0.001)

No.	Authors (Year)	Article title	Country	Primary aim	Sample	Age	Technology/AI	Load monitoring domain	Key findings
				prescripciones of training and estrategias of recovery		profesional)		through analysis discriminante; exclusión estricta data time-outs/intervalos, solo tiempo activo of juego	
17	Sanders GJ, Skodinski S, Peacock CA (2025) [26]	Impact of Early Season Jump Loads on Neuromuscular Performance in Division I Volleyball: Analyzing Force, Velocity, and Power From Countermovement Jump Tests	United States	examine relationship between loads of jump daily and performance neuromuscular during fase inicial of season of volleyball, evaluando cómo variaciones in counts of jumps A diferentes intensidades impactan performance neuromuscular during primer mes of season competitiva	9 athletes women's volleyball División I NCAA	19.4 ± 1.3 years; height: 184.0 ± 7.1 cm (peso no included by restricciones universitarias)	IMU validada VERT 3 (accelerometer 3-ejes, gyroscope, magnetometer) in cresta ilíaca, sistema dual platforms force portable Hawkin Dynamics (1000 Hz) to CMJ matutino; variables jump: duración minutos, counts totales, Jumps 38+ (>38.1cm), Jumps 50+ (>50.8cm); 36 métricas platform force (14 force: RFD frenado, force desplazamiento mínimo, forces mean/pico frenado/propulsión/aterrizaje	Monitoreo 22 días inicio season (17 sessions: 3 matches + 14 prácticas): loads jump daily with IMU during práctica/juego, CMJ dual-platform mañana siguiente (~8am pre-calentamiento, 2 jumps promediados); alineación temporal load día anterior with performance neuromuscular mañana siguiente; analysis correlacional loads jump diferentes intensidades	Hallazgo central: Intensidad jump > volumen total as predictor respuesta neuromuscular. jumps alta intensidad (Jumps 50+): 29/36 métricas platform significativamente correlacionadas (p<0.05). Métricas force: 9/14 correlaciones negativas débiles-moderadas (r=-0.194 A -0.570, p≤0.025) incluyendo RFD frenado (r=-0.478), force desplazamiento mínimo (r=-0.551), forces pico frenado (-0.570) and propulsión (-0)
18	Bartol V,	Influence of the	Eslovenia	analyze symmetry of	30 women (15	Volleyball: 20.1 ± 1.1	Plataforma of force Kistler 9286 AA with	assessment asymmetry	Asymmetry sig. volleyball: Altura CMJ

No.	Authors (Year)	Article title	Country	Primary aim	Sample	Age	Technology/AI	Load monitoring domain	Key findings
	Vauhnik R, Rugelj D (2022) [54]	sport specific training background on the symmetry of the withoutgle legged vertical counter movement jump among female ballet dancers and volleyball players		the CMJ unilateral between pierna dominante and no dominante in volleyball players collegiate female vs. bailarinas of ballet, evaluando cómo the training específico of the deporte influye in patrones of asymmetry neuromuscular and risk of injury	volleyball collegiate, 15 ballet); analysis enfocado in group volleyball	years; height: 175.0 ± 5.3 cm; peso: 68.5 ± 6.5 kg; IMC: 22.4 ± 2.3; training: 4.5 ± 1.0 veces/week	software BioWare and StabDat 3.1; protocolo estandarizado: calentamiento 5 min, determinación dominancia push test, CMJ bilateral/unilateral (dominante/no dominante), 2 jumps familiarización + 3 measurements by tipo, descansos 20s between repeticiones and 2 min between tipos	function neuromuscular as biomarker risk injury and readiness; analysis differences porcentuales pierna dominante vs. no dominante [(dominante-no dominante)/no dominante×100]; identificación déficit contralateral >10% as indicador desequilibrio muscular; aplicación to design training específico que mitigue asymmetrys and optimice Load monitoring domain neuromuscular	difference mean 13.36% (±14.72, range -5.13 A 22.79%) pierna dominante vs. no dominante vs. ballet 4.26% (±10.6%); 67% volleyballistas >10% asymmetry (threshold risk injury). ANOVA 2×2: Efecto principal deporte sig. to energía usada (F_1=16.657, p<0.001, η^2=0.543) and tiempo force maximal aterrizaje (F_1=5.485, p=0.034, η^2=0.587)
19	Karabel, F.; Makaracı, Y. (2025) [59]	"Optimal Recovery Time for Post-Activation Performance Enhance	Turkey	Examine the effects of an acute plyometric bout (RCMJ-30) with different recovery	24 volleyballistas female of level nacional (100% women)	20.83 ± 2.93 years	Plataforma of force Kistler 9260AA6 + software MARS to analysis biomecánico; without componente of AI/ML	PAPE protocol uwithoutg RCMJ-30 to identify the most effective recovery interval (R2, R4, R6, R8) for neuromuscular	Confiabilidad RCMJ-30: ICC = 0.62-0.83 (moderada-buena) to height maximal and mean of jump. CMJ unilateral: Intervalo R8 (8 min) óptimo to height and tiempo of vuelo in both

No.	Authors (Year)	Article title	Country	Primary aim	Sample	Age	Technology/AI	Load monitoring domain	Key findings
		ment After an Acute Bout of Plyometric Exercise on Unilateral Countermovement Jump and Postural Sway in National-Level Female Volleyball players"		intervals (2, 4, 6, 8 min) on unilateral CMJ performance and postural control in national-level female volleyball players.				performance and postural control outcomes.	piernas vs baseline and R2 ($p < 0.01$, sizes of efecto grandes $\eta^2 = 0.24$-0.30). Control postural: Intervalo R6 (6 min) more efectivo to improvement of control postural ($p < 0.05$), especialmente in pierna no dominante with efectos grandes (η^2 up to 0)
20	Sanders, G.J.; Skodinski, S.; Cabarkapa, D.V.; Howard, M.; Cabarkapa, D.; Peacock, C.A. (2024) [45]	"Positional Differences in Jump Loads and Force and Velocity Metrics Throughout A 16-Week Division I Volleyball Season"	United States	quantify loads totales and of alta intensidad of jumps (>38.1 cm and >50.8 cm) and evaluate performance neuromuscular through métricas of force and velocity of CMJ by posición during	12 athletes female División I NCAA (100% women)	19.6 ± 1.3 years	Microsensores VERT 3 (IMU tri-axial) + platforms of force duales Hawkin Dynamics (1000 Hz) + analysis statistical with models of efectos mixtos; without componente of AI/ML	Monitoreo longitudinal 16 weeks of loads of jump diferenciadas (totales, jumps 38+, jumps 50+) with assessment neuromuscular bi-weekly through CMJ to analysis of fatigue and adaptaciones positionales	difference positionales significativas ($p \leq 0.001$): MB acumularon more loads alta intensidad (jumps 38+: 65.4±39.2; jumps 50+: 39.5±32.7) vs. S (jumps 38+: 19.0±16.6; jumps 50+: 0.4±0.8). performance CMJ diferenciado: MB greater height (36.1±6.4 cm), profundidad (-41.7±6.4 cm) and velocidades propulsión (pico: 2.75±0.22 m/s); S greater force braking RFD (7839±2617 N/s) and forces propulsión pese A loweres loads

No.	Authors (Year)	Article title	Country	Primary aim	Sample	Age	Technology/AI	Load monitoring domain	Key findings
				season completa of 16 weeks					
21	Nikolaidou, M.-E.; Sotiropoulos, K.; Barzouka, K. (2023) [21]	"Postural balance ability and vertical jumping performance in female veteran volleyball athletes and non-athletes"	Greece	investigate capacity of equilibrio postural and performance of jump vertical in athletes veteranas of women's volleyball (activas and retiradas) comparadas with no-athletes; examine efectos of restricción visual in equilibrio and associations between equilibrio and performance of jump	81 women of mediana Age (66 athletes veteranas volleyball + 15 controles no-athletes, 100% women)	50 ± 5 years (activas: 50.4±4.4; retiradas: 51.1±5.3; control: 50.2±4.4)	Plataforma of force Wii (100 Hz, 24-bit) + analysis MATLAB personalizado to métricas CoP and CMJ; without componente of AI/ML	assessment of equilibrio postural estático (unipodal/bipodal, ojos abiertos/cerrados) and performance CMJ as biomarkeres of function neuromuscular; analysis of adaptaciones A training systematic A largo plazo and retención of capacityes after diferentes estados of actividad	Equilibrio postural diferenciado: Solo in unipodal, athletes (activas and retiradas) greater range oscilación mediolateral vs. control (p<0.001), interpretado as estrategia adaptativa to exploración postural específica of the deporte. performance explosivo superior: athletes activas and retiradas vs. control in height CMJ (15.9±3.1 cm and 14.5±4.7 cm vs. 8.1±3.3 cm, p<0.001), power mean (17.0±2.6 and 15.9±3.1 vs. 13.1±2
22	Bellinger, P.M.; Newans, T.; Whalen	"Quantifying the Activity Profile of Female Beach	Australia	determine perfiles of output externo in volleyball players	20 players volleyball playa female (100% women)	Adultas: 27.4 ± 2.6 years; U23: 19.3 ± 2.4 years	GPS VX Sport VX110 Log (10 Hz GPS + accelerometer tri-axial 100 Hz) + software Visuallex Sport; models lineares mixtos	Monitoreo continuo during 60 matches of torneos oficiales with analysis of distancia	difference by level competitivo: Adultas greater distancia relativa in zonas velocity 2 (1.0-1.99 m/s: 42.0% vs 37.9%, p<0.001) and

No.	Authors (Year)	Article title	Country	Primary aim	Sample	Age	Technology/AI	Load monitoring domain	Key findings
	, M.; Minahan, C. (2021) [24]	Volleyball Tournament Match-Play"		playa female during competition of torneos; evaluate efectos of level competitivo, margen of puntuación and alteraciones intra-partido			(lme4/afex in R); without componente of AI/ML	total/relativa, velocidades by zonas, accelerations/desaceleraciones categorizadas, efectos contextuales (level competitivo, margen puntuación, progresión temporal) to caracterización integral of demandas competitivas	zona 3 (≥2.0 m/s: 10.9% vs 9.4%, p=0.003) vs U23; U23 greater distancia zona 1 (0.0-0.99 m/s: 52.5% vs 46.3%, p<0.001).Variaciones intra-partido: Set 1 > Set 2 in distancia relativa (37.08±0.73 vs 35.54±0.75 m/min, p=0.001), aceleración/desaceleración mean (0.77±0.02 vs 0.75±0
23	Kupperman, N.; Curtis, M.A.; Saliba, S.A.; Hertel, J. (2021) [3]	"Quantification of Workload and Wellness Measures in A Women's Collegiate Volleyball Season"	United States	quantify loads internas and externas completadas by athletes of volleyball collegiate female during season competitiva; explorar differences positionales in ambos tipos of measures and evaluate	11 players volleyball collegiate División I NCAA (100% women)	19.36 ± 1.27 years	Acelerómetros Catapult Clearsky T6 (100 Hz tri-axial) + platform AthleteReady to wellness + RPE Borg CR-10; analysis Python 3.8.0 with statistical descriptiva e inferencial; without componente of AI/ML	Monitoreo longitudinal 101 días (55 prácticas + 30 juegos) with métricas external load (PlayerLoad, COD, accel/decel, jumps categorizados, RHIE), internal load (RPE), wellness multidimensional (5 dimensiones, escala 7 puntos); analysis by períodos season and diferenciación	Patrón load práctica > juego: Todas the métricas acelerométricas greater in prácticas vs juegos (PL: 388.3±128.5 vs 348.4±145.9; jumps: 90.9±51.2 vs 81.1±49.8; COD: 247.5±121.7 vs 229.4±124.8; todos p<0.0001), explicado by naturaleza rotacional of the volleyball. Efectos temporales in wellness: Deterioro sig. during juego of conferencia vs preseason in stress (p=0.002), dolor muscular (p=0)

No.	Authors (Year)	Article title	Country	Primary aim	Sample	Age	Technology/AI	Load monitoring domain	Key findings
				tendencias longitudinales				positional	
24	Fuchs, P.X.; Mitteregger, J.; Hoelbling, D.; Menzel, H.-J.K.; Bell, J.W.; von Duvillard, S.P.; Wagner, H. (2021) [47]	"Relationship between General Jump Types and Spike Jump Performance in Elite Female and Male Volleyball players"	Austria (colaboración internacional: Italy, Brazil, United States)	analyze relaciones between jumps generales (SJ, CMJ, CMJA) and jump of remate específico (VSJ); identify factors more apropiados to assessment of performance VSJ; providesr models prácticos to prediction improvementda of height VSJ by sexo	30 volleyballistas of elite nacional (15 women + 15 men, 50% female)	Women: 19.9 ± 3.5 years; men: 22.7 ± 4.3 years	13 cameras Vicon MX-13 (250 Hz) + 2 platforms AMTI (2000 Hz) to analysis biomecánico 3D; models regression stepwise-forward with analysis correlacional; without componente AI/ML avanzada	assessment multidimensional capacityes neuromusculares through analysis cinemático-cinético of jumps generales and specific; desarrollo of models predictive to optimization of assessment específica of the volleyball with variables RFD, impulso, velocidades angulares	Correlaciones altas between jumps: VSJ correlacionó fuertemente with todos the jumps generales in ambos sexos (women: SJ r=0.88, CMJ r=0.88, CMJA r=0.82; men: SJ r=0.80, CMJ r=0.85, CMJA r=0.75, todos p<0.001). Limitations models simples: models usando solo height of jump general explicaron únicamente 52-76% of varianza in height VSJ, insuficiente to prediction precisa
25	Sharma, M., & Singh, A. (2025) [35]	"A cross-sectional study on gender & sports specific biomecha	India	evaluate parameters cinéticos (force, velocity, power, tiempo)	96 athletes collegiate indios (mixto with analysis desagregad	18-24 years (volleyball: ~19.8 years mean)	Plataforma of force Kistler Quattro Jump Type 9290DD (500 Hz, range 10 kN) + software MAR v5.2.1.237 to proceswithoutg; analysis statistical SPSS	assessment biomechanical transverse of function neuromuscular through CMJ estandarizado (3	difference of género significativas in volleyball: men superiores A women in greatería of parameters CMJ (RMAXF: 232.76±11.34 vs

No.	Authors (Year)	Article title	Country	Primary aim	Sample	Age	Technology/AI	Load monitoring domain	Key findings
		nical analysis of countermovement jump force, velocity, power and time parameters in Indian players"		during CMJs in players masculinos and females of cuatro deportes (fútbol, atletismo, volleyball, críquet) in población universitaria india; examine efectos of género, groups deportivos and sus interacciones in variables of performance neuromuscular	o by sexo; group volleyball *n* = 24 with comparaciones específicas masculino vs. female)		v26.0 with t-test independiente and MANOVA; without componente of AI/ML	repeticiones, without braceo) with 16 parameters cinéticos detallados to caracterización of capacityes explosivas, diferenciación by género and deporte, and establecimiento of benchmarks specific to optimization of performance and management of readiness	195.50±8.91 %BW; AMF: 1316.08±192.05 vs 1032.05±178.21 N; VTOV: 2.57±0.12 vs 1.92±0.14 m/s; JHTOV: 0.32±0.02 vs 0.18±0.02 m; RMP: 47.96±4.0 vs 32.09±1.66 W/kg; todos p<0.001), excepto in POT donde women mostraron valores greater (0.32±0.02 vs 0.29±0.02 s, p<0
26	Liao, L.; Li, J. (2022) [17]	Research on Effect of Load Stimulation Change on Heart Rate Variability of Women Volleyball	China	Explore the effects of different training-load stimuli on HRV and central nervous system-related parameters	125 volleyballistas female of elite (Superliga China + team nacional)	21.3 ± 3.1 years; experience: 7.9 ± 2.7 years	Sistema OmegaWave Sport Technology (ECG/EEG no invasivo, sensores Bluetooth, módulo TGAM); XGBoost + algorithm assessment distancia improvementdo; sistema M-HMI with machine learning	Monitoreo multidimensional HRV + function SNC during 3 etapas diferenciadas of training invernal; assessment dentro of 1h post-session; correlation load	SDNN índice more sensible to estado real of athletes (aumenta gradualmente with tiempo of ejercicio); LF n.u. more sensible in dominio frecuencial (patrón descenso-recovery); índice estabilidad respuesta SNC disminuye

No.	Authors (Year)	Article title	Country	Primary aim	Sample	Age	Technology/AI	Load monitoring domain	Key findings
		athletes		in elite female volleyball players over A 2-year longitudinal follow-up; propose A classification method based on workload and recovery markers.				planificada-respuesta interna	significativamente in etapa mean (F=3.757, p=0.026) and se recupera in tardía; SDNN and LF n.u. impacto sig. in tiempo reacción mean
27	Lupo, C.; Ungureanu, A.N.; Brustio, P.R. (2020) [40]	Session-RPE is A valuable internal loading evaluation method in beach volleyball for both genders, elite and amateur players, conditioning and technical sessions, but limited for tactical	Italy	Verificar correlation between métodos session-RPE and Edwards to quantify internal load in volleyball playa considerando género, level competitivo (elite/amateur) and tipo of session (acondicionamiento, técnicas, tácticas/jue	36 players volleyball playa (18 women: 9 elite + 9 amateur; 18 men: 9 elite + 9 amateur). Monitored sessions: 386 individuales (elite: 197, amateur: 189)	Élite: women 22±3 years, men 23±1 years; Amateur: women 26±4 years, men 28±4 years	Polar Team System (FC 1 Hz, transmisión inalámbrica, memoria interna); session-RPE (escala CR-10 modificada Foster); método Edwards (zonificación FC with HRmax estimada 220-Age); without AI/ML	validation concurrent of session-RPE vs. Edwards to ITL; analysis correlacional by subgroups (género×level×tipo session); assessment diferencial ITL between tipos of training	Correlaciones session-RPE vs Edwards: Género female r=0.75 (IC95%: 0.68-0.81, p<0.001); masculino r=0.76. by level: elite r=0.77, amateur r=0.75. by tipo session: acondicionamiento r=0.75 (muy grande), técnicas r=0.61 (grande), tácticas/juego r=0.36 (moderada). ITL Edwards diferencial: técnicas < acondicionamiento ≈ tácticas/juego (p<0.001, ES=0.7):

No.	Authors (Year)	Article title	Country	Primary aim	Sample	Age	Technology/AI	Load monitoring domain	Key findings
		training and games		go)					
28	Cabarkapa, D.V.; Cabarkapa, D.; Fry, A.C. (2024) [63]	Starters vs. non-starters differences in vertical jump force-time metrics in female professional volleyball players	United States (athletes europeas)	examine differences in métricas force-tiempo of the CMJ between titulares and suplentes in players profesionales of women's volleyball to identify si the performance neuromuscular difference the estatus competitivo	19 players profesionales women's volleyball SuperLeague europea (titulares *n* = 9: >75% juegos iniciados; suplentes *n* = 10)	21.6 ± 2.8 years; height: 181.5 ± 7.0 cm; peso: 74.2 ± 8.3 kg	Plataforma of force uni-axial ForceDecks Max (VALD Performance, 1000 Hz); analysis 19 métricas force-tiempo CMJ (fases excéntrica/concéntrica); algorithms propietarios VALD to cálculo variables biomechanical complejas; without AI/ML	assessment function neuromuscular tren inferior through analysis biomecánico detallado CMJ; diferenciación capacityes explosivas by level competitivo; aplicación to monitoreo readiness and estado neuromuscular	Hallazgo central: without differences statisticalmente significativas between titulares and suplentes in todas the 19 métricas force-tiempo evaluadas (p>0.05). Tamaños of efecto: Pequeños-moderados (g=0.053-0.683), excepto profundidad contramotion (g=0.804, grande). Valores representativos: Altura jump titulares 29.7±5.3cm vs suplentes 29.1±3.1cm; RSI-modificado 0.42±0.12 vs 0.38±0.07
29	Tsarbou, C.; Liveris, N.I.; Tsimeas, P.D.; Papageorgiou, G.; Xergia, S.A.;	the effect of fatigue on jump height and the risk of knee injury after A volleyball training	Greece	investigate the efecto of the fatigue inducida by A juego of training of volleyball on the height of jump and the risk of injury of the	13 players universitarias of women's volleyball (estudiantes Educación Física Universidad of	19.3 ± 1.1 years (range 18-21); height: 1.68 ± 0.04 m; peso: 67.0 ± 12.3 kg; training: 4.2 ± 1.1 sessions/week	Sistema Bosco Ergojump (jump vertical); analysis video 2D with cameras Panasonic HC-V770/Sony HDR-CX625; software Kinovea v0.8.26; LESS (Landing Error Scoring System) with 17 ítems assessment; escala Borg	assessment pre-post juego volleyball 60min (3 sets) with measurement multidimensional fatigue: biomarkeres objectives (height jump vertical, técnica aterrizaje	Inducción fatigue sig.: RPE pre-juego 6.5±0.4 vs post-juego 10.2±0.6 (t_{12}=14.05, p<0.001, d=3.9). Deterioro function neuromuscular: Altura jump pre-juego 26.2±2.3cm vs post-juego 24.9±2.2cm (t_{12}=2.55, p=0.026, d=0.6, reduction 5%). increase risk injury

No.	Authors (Year)	Article title	Country	Primary aim	Sample	Age	Technology/AI	Load monitoring domain	Key findings
	Tsiokanos, A. (2021) [53]	game: A pilot study		LCA through assessment multidimensional of biomarkeres objectives and subjetivos	Tesalia)		RPE (6-20 puntos); without AI/ML	LESS) and subjetivos (RPE); analysis deterioro neuromuscular and risk injury LCA in contexto real training	LCA: LESS pre-juego 5.8±1.0 vs post-juego 6.3±1.1 (t_{12}=2.21, p=0.047, d=0.7). Deterioro técnica aterrizaje
30	Holtgeerts, R.N.; Gann, J.; Jung, H.C.; Hey, W. (2022) [60]	the Impact of Recovery Time on Performance in Division I Collegiate Beach Volleyball players	United States (colaboración with Korea of the Sur)	examine efectos of diferentes tiempos of recovery (24h vs 48h) on performance físico, tactical and perceptual in players universitarias División I of volleyball playa through assessment multidimensional in contexto real of juego	10 players universitarias volleyball playa female División I NCAA	20.2 ± 1.23 years; height: 173.5 ± 5.99 cm; peso: 68.9 ± 6.93 kg; % grasa corporal: 24.4 ± 3.68; jump vertical: 47.6 ± 1.23 cm	Dispositivo VERT (sensor inercial validado to height/count jumps, load of trabajo); monitores FC Polar Electro; PRS (0-10), escala visual analógica calidad sleep (10 cm), Omni RPE (0-10); analysis statistical SPSS v26 with ANOVA measures repetidas; without AI/ML	Design cruzado randomized counterbalanced comparing recovery 24h vs 48h between sessions volleyball playa; assessment pre-post multidimensional: CMJ in arena, agilidad 5-10-5, performance during juego (height jump pico/mean, count jumps), drills tactical specific (4-Corner, Defensive Moves, Serve-Receive), matches full NCAA, measures perceptual (PRS, sleep, RPE)	performance físico diferencial: CMJ mejoró significativamente with recovery 24h (46.8±6.82 vs 49.2±8.04 cm, p=0.050, d=0.32). Agilidad 5-10-5 mejoró significativamente with recovery 48h (5.3±0.30 vs 5.2±0.32s, p=0.014, d=0.32). performance during juego estable: without differences significativas in height jump pico/mean, count jumps during matches between condiciones recovery
31	Vavassori, R.; Moren	the Perception of	Spain	Conocer percepciones of players	22 players universitarias	19.41 ± 1.74 years (range 17-23); 18	questionnaire well-being (5 ítems, escala Likert 1-5, Excel/Google	Monitoreo season completa (sept-mayo) with	Impacto herramientas monitoreo: questionnaire well-being generó

No.	Authors (Year)	Article title	Country	Primary aim	Sample	Age	Technology/AI	Load monitoring domain	Key findings
	o, M.P.; Ureña Espa, A. (2023) [42]	Volleyball Student-athletes: Evaluation of Well-Being, Sport Workload, players' Response, and Academic Demands		universitarias of women's volleyball on herramientas of monitoreo (questionnaire well-being, sRPE, CMJ) to assessment of well-being, loads of trabajo, respuestas A loads and demandas académicas through analysis cualitativo fenomenológico	women's volleyball (11 primer team nacional, 9 team youth U19 regional, 2 ambos teams)	estudiantes universitarias/moreter, 4 bachillerato	Drive); sRPE (escala CR-10 Foster, cálculo automático load trabajo); CMJ (app MyJump 2 validada, iPhone 5s 120fps, 2 veces/week); platform digital integrada; analysis cualitativo temático reflexivo; without AI/ML	assessment multidimensional: questionnaire well-being diario, sRPE post-session, CMJ bi-weekly, registro demandas académicas; integración data real time through platform digital; betweenvistas semi-estructuradas to the final of season	conciencia (100% athletes): 50% neutral, 50% positiva; factors principales: fatigue (59%), demandas académicas (50%), problemas personales (41%). sRPE: Conciencia 95% athletes; 73% desarrolló autorregulación/autodemanda/autoassessment; factors: fatigue (59%), estado ánimo (18%), demandas académicas (14%). CMJ:Respuesta positiva 86% (motivación/competition)
32	Villarejo-García, D.H.; Moreno-Villanueva, A.; Soler-López, A.; Reche-Soto,	Use, Validity and Reliability of Inertial Movement Units in Volleyball: Systematic Review of the Scientific	Spain (Universidad of Murcia, Universidad Isabel I)	examine the validez and confiabilidad of múltiples dispositivos IMU to medir height of jump in volleyball, determining the grado of	21 studies included (*n* = 5-115 sujetos by study); 90.47% modalidad indoor, 9.53% playa; 57.17% studies with men,	range 16.1-27.6 years in studies included; predominancia athletes elite (90.47%) and level local (9.37%)	IMUs: Vert (57.14% more usado), Catapult, Suunto, Shimmer, Blast, Zephyr BioHarness; colocación predominante cresta ilíaca (80.95%); without componente AI/ML significant	Monitoreo external load through count jumps (77% studies) and height jumps (63% studies); algorithms Player Load in 32% studies; correlaciones with internal load (RPE)	Validez concurrente excelente: Conteo jumps 95-99% accuracy vs. observación visual experta; correlaciones r=0.83-0.97 between IMU and gold standard. Confiabilidad limitada: Altura jumps with errores sistemáticos, subestimación típica 2.5-4.1 cm; límites acuerdo amplios (-6.1 A 9.8 cm)

No.	Authors (Year)	Article title	Country	Primary aim	Sample	Age	Technology/AI	Load monitoring domain	Key findings
	P.; Pino-Ortega, J. (2023) [6]	Literature		validez and confiabilidad of estos dispositivos comúnmente utilizados by coaches	23.77% women, 19.06% ambos sexos				
33	Tometz, M.J.; Jevas, S.A.; Esposito, P.M.; Annaccone, A.R. (2022) [13]	Validation of Internal and External Load Metrics in NCAA D1 Women's Beach Volleyball.	United States (Texas Christian University, University of Texas at Arlington)	determine the validez of métricas of internal load and externa in volleyball playa female NCAA División I, estableciendo relaciones between TRIMP (Edward's), sRPE Load and distancia recorrida through technologies of monitoreo no invasivo	13 players NCAA D1 volleyball playa female (100% women); 578 observaciones individuales of 51 sessions of team during 15 weeks preseason (38 prácticas, 11 matches, 2 acondicionamientos specific)	20.3 ± 1.4 years (range 18-22 years); height: 176.2 ± 4.3 cm; peso: 67.8 ± 5.7 kg	Polar Team Pro (monitor heart rate integrado with GPS), Kestrel 5400 Heat Stress Tracker to condiciones ambientales, TeamBuildr app to sRPE; without componente AI/ML pero establece fundamentos to algorithms predictive	validation cruzada métricas internal load (TRIMP Edward's, sRPE Load) vs. externa (distancia recorrida GPS) through analysis correlacional and models of regression predictiva during monitoreo longitudinal 15 weeks	Concurrent validity confirmed: very strong, significant correlations ($p < 0.01$) between TRIMP and sRPE load ($r = 0.81$, 95% CI 0.78–0.84), TRIMP and distance ($r = 0.78$, 95% CI 0.74–0.81), and sRPE load and distance ($r = 0.82$, 95% CI 0.79–0.85).
34	Marzano-Felisatti, J.M.; Pino-Ortega,	Validation of the WIMU PRO™ Device for Jump	Spain (Universidad of Valencia, Universidad of	evaluate the validez of the dispositivo WIMU PRO™ to detección	11 players volleyball playa (6 women, 5 men); 1,481	Women: 27±3 years; men: 28±3 years (población adulta	WIMU PRO™ multi-sensor (RealTrack Systems): 4 accelerometers triaxiales, 3 gyroscopes triaxiales, 1 manómetro	validation detección automática jumps to cuantificación external load through analysis	Sensibilidad excelente general: 96.29% (WIMU PRO™: 1,426 vs Observación: 1,481 jumps). difference género significativas: men

No.	Authors (Year)	Article title	Country	Primary aim	Sample	Age	Technology/AI	Load monitoring domain	Key findings
	J.; García-de-Alcaraz, A.; Portillo, J.; Guzmán-Luján, J.F.; Priego-Quesada, J.I. (2025) [16]	Detection in Beach Volleyball: A Gender-Based Analysis during Official Competitions	Murcia, Universidad of Almería, Universidad of Castilla-the Mancha)	automática of jumps in volleyball playa and determine preliminarmente si the género, individualidad of the player o acción técnica asociada to the jump influyen in the accuracy of the data during competiciones oficiales	jumps analizados during 42 sets oficiales; women: 27±3 years, 65.9±3.9 kg, 1.73±0.03 m; men: 28±3 years, 80.5±4.3 kg, 1.84±0.07 m; level competitivo Tier 3-4	competitiva organizada)	triaxial, GPS, UWB; algorithm automático detección jumps (velocity despegue ≥1.4 m/s^2, tiempo vuelo ≤1500 ms, force impacto ≥2G); validation vs analysis observacional video HD (GoPro Hero 4) as gold standard; software LINCE PLUS and SPRO	jump-by-jump during competition oficial; categorización by tipo jump (Saque, Remate, Bloqueo, Otros) with analysis diferencial by género e individualidad of the player to optimization monitoreo personalizado	97.20% vs Women 94.56%; men greater falsos positivos (2.5% vs 0.6%), women greater falsos negativos (5.4% vs 2.8%). Variabilidad inter-player notable: Women range 91.06%-98.08% vs men 95.02%-98.40%. Especificidad by tipo jump:"Otros" 99.10%, "Bloqueo" 97.25%, "Remate" 95
35	Lima, R., Silva, B., Gracinda, A., Silva, A.F., Pereira, J., Silva, R.M., Clemente, F.M. (2024) [38]	Week-to-week variations of internal and external intensity measures in professional women volleyball players	Portugal	(i) analyze the relationship between variaciones weekles of ACWR, monotonía of training (TM), tensión of training (TS), sRPE and No. of jumps; (ii) Explorar the	10 players profesionales of women's volleyball (Primera División portuguesa: 2 armadoras, 3 middle blockers, 3 atacantes externas, 2 opuestas)	24.1 ± 6.12 years; 66.48 ± 6.85 kg; 175.4 ± 6.52 cm	IMU Vert® Classic (accelerometers, gyroscopes, magnetometers tri-axiales) with transmisión Bluetooth A MyVert Coach; sRPE Borg CR-10; accuracy validada: error medio -0.945 pulgadas vs. métodos gold standard; without AI/ML, métricas automatizadas	Externa: No./height of jumps by session vía IMU; Interna: sRPE (RPE × duración session); Métricas derivadas: ACWR (1 sem aguda/4 sem crónica), monotonía (TM), tensión training (TS) calculadas weeklmente	Correlaciones moderadas between variaciones weekles of ACWR/TM/TS and sRPE/jumps; sRPE mostró greater sensibilidad (greater % variación) que No. of jumps; correlaciones significativas sem. 2 ($r=0.852$, $p<0.001$) and sem. 7 ($r=0.813$, $p<0.05$); patrón temporal "W" in aplicación loads; inconsistencias sRPE-jumps (sem. 7-9) sugieren retraso

No.	Authors (Year)	Article title	Country	Primary aim	Sample	Age	Technology/AI	Load monitoring domain	Key findings
				association between measures of intensidad interna and externa A través of weeks					perceptual
36	Akyildiz, Z., de Oliveira Castro, H., Çene, E., Laporta, L., Parim, C., Altundag, E., Akarçeşme, C., Guidetti, G., Miale, G., Silva, A.F., Nobari, H., Clemente, F.M. (2022) [10]	Within-week differences in external training load demands in elite volleyball players	Turkey (colaboración internacional: Brazil, Spain, Italy, Portugal, Irán, Rumania)	Analyze within-week differences in external training-load intensity across microcycle days (MD-5 to MD+1), considering playing positions in elite women's volleyball.	14 volleyball players female of elite (team campeón mundial of clubes, Liga Turca): Atacantes externas izquierdas (LH), atacantes externas derechas (RH), middle blockers (MB), armadoras (S), liberos (L)	22 ± 0.9 years; height: 195.1 ± 7.6 cm; peso: 71.4 ± 6.3 kg	Sistema LPS KINEXON GMBH: 12 antenas with accuracy milimétrica (calibración taquímetro), validado vs. sistema VICON (error típico estandarizado 0.06-6.0%, coeficiente variación 0.7-7.3%); Variables monitoreadas: distancia total LPS, jumps LPS, accelerations/desaceleraciones categorizadas, distancia metabólica alta (HMLD), velocity maximal; without AI/ML: analysis statistical convencional (ANOVA, Tukey HSD, Bonferroni)	External: continuous LPS monitoring during training and matches (normalized by session duration); derived metrics included ACWR (Rolling Average and EWMA), monotony, strain, and standardized loads. Internal: sRPE (RPE × duration).	difference by microciclo: Significativas ($p<0.05$) with sizes of efecto moderados-grandes (η^2=0.084-0.800) in todas the variables excepto tensión, ratio Acc/Dec and load mean aguda (RA); Patrón temporal: reduction progresiva of loads aproximándose to the día of partido (MD and MD-1 < MD-2 A MD-5); difference positionales: Centrales (MB) acumularon loweres loads vs. otras positions
37	Rebelo,	Beyond	Portugal	Proporciona	review	range	Mapeo systematic of	Mapeo	Hallazgos of the mapeo

No.	Authors (Year)	Article title	Country	Primary aim	Sample	Age	Technology/AI	Load monitoring domain	Key findings
	A., Pereira, J.R., Nakamura, F.Y., Valente-dos-Santos, J. (2024) [8]	the Jump: A Scoping Review of External Training Load Metrics in Volleyball	(colaboración internacional)	r A visión integral of the measurement of load of training externa (ETL) in volleyball, systematically map métricas and technologies utilizadas, identify gaps in the knowledge actual (especialmente beyond of the plane sagittal and to positions específicas as liberos) and ofrecer perspectivas valiosas to investigadores and profesionales	sistemática exploratoria: 18 studies included with populations diverse: masculinas and female; leveles elite (67%), collegiate (22%), youth (11%); sizes sample of 5-24 participants by study; ages 16-30.5 years; positions: outside hitters, setters, middle blockers, opposites (39% studies), liberos (22% studies)	diverso según studies included: 16-30.5 years (no aplica Age única by ser review)	technologies no invasivas: IMU tri-axiales predominantes (Vert Classic MyVert, Catapult Vector S7/T6) with accelerometers, gyroscopes, magnetometers; Sistema LPS KINEXON to ambientes indoor; analysis of video multicámara; combinaciones IMU+video; Procesamiento algorithmic:PlayerLoad, ACWR (RA and EWMA), métricas of monotonía and tensión; without AI/ML directa	comprehensivo of métricas ETL: Predominio plane sagittal (94% studies): count/height/load of jumps, jumps >50cm; métricas limitadas planes frontal/transverse: distancia total, accelerations/desaceleraciones, load metabólica alta (HMLD), velocity, PlayerLoad; gap identificada:subrepresentación of motions multidireccionales and métricas of extremidad superior (arm swing)	systematic: gap sustancial in measurements ETL beyond of the plane sagittal (solo 6% studies incluyen planes frontal/transverse); subrepresentación crítica of posición líbero (solo 22% studies); predominio tecnológico IMU pero limitations of estandarización; Asociaciones identificadas: lower count of jumps and greater variabilidad ETL preceden injuries
38	Marott	Correlatio	Italy	evaluate the	60 athletes	women's	EMG superficial	Focus of	difference of género

No.	Authors (Year)	Article title	Country	Primary aim	Sample	Age	Technology/AI	Load monitoring domain	Key findings
	a, N., Demeco, A., Moggio, L., Isabello, L., Iona, T., Ammendolia, A. (2020) [55]	n between dynamic knee valgus and quadriceps activation time in female athletes		correlation between the increase of the valgo dinámico of knee and the reduction of the ratio mediolateral of the cuádriceps in reclutamiento and timing during A motion que somete to the ligamento cruzado anterior (LCA) A stress, comparing athletes masculinos and females	totales (30 men, 30 women) of tres deportes: subsample volleyball: 20 athletes elite (10 women: 23.9±3.3 years; 10 men: 26±3.9 years); also incluye ballet clásico youth (*n* = 20) and fútbol elite (*n* = 20)	volleyball: 23.9 ± 3.3 years (elite); range total of the study: 13.4-26.1 years	wireless: FREEEMG1000 (BTS bioengineering, 1000 Hz) with 4 electrodos to rectus femoris (RF), vastus medialis (VM), semitendinosus (ST), biceps femoris (BF); Sensor inercial: G sensor (BTS) to withoutcronización timing muscular-contacto with suelo; Software integrado: EMG Analyzer (BTS) to analysis temporal; Analysis video 2D: Kinovea v0.8	assessment neuromuscular and biomechanical: Drop-fall test from platform 32cm as tarea estandarizada que estresa the LCA; Métricas of readiness: timing of pre-activación muscular (lower tiempo = activación tardía) and ángulo of valgo dinámico as indicadores of control neuromuscular; Aplicabilidad limitada: more orientado A screening of risk lesional que A monitoreo directo of load of training	significativas: Women mostraron activación more tardía of the vastus medialis vs. men in volleyball (207.7±52.4 ms vs. 402.4±47.3 ms - donde lower tiempo indica activación tardía); Valgo dinámico aumentado: Women presentaron greater DVKa (6.1±0.9°) vs. men (3.1±0.9°) in volleyball; correlation fuerte: r=0.79 (t=9.84, p<0.05) between activación tardía of the VM and greater DVKa; R^2=0.639 (62)
39	Rebelo, A., Teixeira, D.S., Martinho, D.V., Valente	From data to action: A scoping review of wearable technologies and biomecha	Portugal (colaboración multi-institucional: Universidade de Lusófona,	evaluate the uso actual of technologies in sport settings to adaptation of the training and	review sistemática exploratoria: 21 studies primarios included with	range diverso según studies included: 16-42 years (no aplica Age única by ser	Systematic mapping of non-invasive technologies: triaxial accelerometers were most common (67% of studies), followed by GPS (52%) and force platforms (33%); device	Mapeo comprehensivo of aplicaciones: Player Load identificado as métrica central to individualización of loads según	Hallazgos of the mapeo systematic: Uso extendido of technologies wearables to monitoreo aim of the performance and orientación of decisiones basadas in evidencia; Player Load as

No.	Authors (Year)	Article title	Country	Primary aim	Sample	Age	Technology/AI	Load monitoring domain	Key findings
	-dos-Santos, J., Coelho-e-Silva, M.J. (2023) [18]	nical assessments informing injury prevention strategies in sport	Sporting CP, Universidad of Coimbra, ITI/LARSYS)	injury prevention; systematically map the literatura existente, identify conceptos and temas clave, and highlight gaps in the research to guide studies future in the field of technologies wearables and evaluaciones biomechanical	populations diverse: 10 studies masculinos exclusivos (48%), 8 studies females exclusivos (38%), 3 studies mixtos (14%); range etario 16-42 years; leveles collegiate (48%), profesionales (29%); subsample volleyball: 5 studies (24% of the total) representando aplicaciones específicas in women's volleyball and mixto	review sistemática)	types included IMUs and positioning systems.	deporte, posición of the player and demandas físicas específicas; Aplicaciones diferenciadas by deporte	métrica central: presente in 48% of studies, permite personalización of loads of training según demandas específicas of the deporte, posición and actividades; Aplicaciones preventivas validadas
40	Cardoso, A.S., Klein,	Heart rate profile and heart	Brazil (Universidade	Realizar A review sistemática	review sistemática with meta-	mean global of ambos sexos: 22 ±	Non-invasive monitoring of cardiovascular	Monitoreo fisiológico integral of	Perfil cardiovascular característico: FC rest elevada to level fitness of

No.	Authors (Year)	Article title	Country	Primary aim	Sample	Age	Technology/AI	Load monitoring domain	Key findings
	L., Harden, L., Kruel, L.F.M., Costa, R.R. (2022) [39]	rate variability in volleyball athletes: A systematic review with meta-analyses	Federal do Rio Grande do Sul, Porto Alegre)	with meta-analysis to establecer the perfil característico of athletes of volleyball respecto A variables of heart rate (FC rest, FC maximal, FC training, FC competition) and variabilidad of the heart rate (VFC) in dominio temporal, providesndo valores of referencia to interpretación of data of monitoreo fisiológico	analysis: 32 studies included representando 477 athletes totales (325 men, 152 women); Características demográficas: Age mean 22±3.1 years (range 16-27 years), peso corporal 76±1.5 kg, IMC 23±0.3 kg/m²; experience mínima 2 years, volumen training 6-20 horas/week	3.1 years (range of studies included: 16±0.5 A 27±4.5 years)	parameters: Polar heart-rate monitors (S610i, S810, Accurex Plus, Electro), electrocardiography (ECG lead DII), telemetry systems, blood-pressure monitors, and metabolic analyzers.	internal load: FC as indicador of intensidad of ejercicio adaptado A características intermitentes of the volleyball; Perfil FC establecido: FC rest (66±2.5 bpm), FC maximal (184±1.3 bpm), FC training (150±12 bpm), FC competition (154±5.5 bpm); VFC to assessment autonomic	athletes (66±2.5 bpm, similar población general vs. athletes aeróbicos elite 28-40 bpm); FC maximal comparable otros deportes of team (184±1.3 bpm, similar fútbol 187±8 bpm, superior baloncesto 165-171 bpm); Demanda competitiva diferenciada: FC competition (154±5)
41	Cabarkapa, D., Cabarkapa, D.V.,	Inter-limb asymmetries in professional male	United States (colaboración internacio	examine differences in asymmetrys inter-	70 athletes profesionales masculinos of ligas	Volleyball: 24.4 ± 3.9 years; Baloncesto: 25.7 ± 3.3	Plataforma of force dual uni-axial portable: ForceDecks Max (VALD Performance, Brisbane, Australia) with	Neuromuscular assessment for readiness and injury prevention:	difference significativas in todas the métricas of asymmetry: CMJ vs. SLJ with sizes of efecto moderados-grandes

No.	Authors (Year)	Article title	Country	Primary aim	Sample	Age	Technology/AI	Load monitoring domain	Key findings
	Fry, A.C. (2025) [58]	basketball and volleyball players: bilateral vs. unilateral jump comparison	nal: University of Kansas, D2 Lab Serbia, Singidunum University Serbia)	extremidades between jump vertical with contramotion (CMJ) bilateral and jump unilateral (SLJ) utilizando A sistema of platforms of force portable innovador in athletes profesionales of baloncesto and volleyball, to determine si estas tests pueden usarse intercambiablemente in the assessment of asymmetrys neuromusculares	internacionales of elite (NBA, EuroLeague, SuperLeague): subsample volleyball: 35 players (Age: 24.4±3.9 years; height: 196.8±5.2 cm; peso: 93.8±16.5 kg); subsample baloncesto: 35 players (Age: 25.7±3.3 years; height: 200.8±7.1 cm; peso: 93.8±16.5 kg); todos activos without injuries musculoesqueléticas 6 meses previos	years	frecuencia muestreo 1000 Hz; Software analysis: ForceDecks Max to derivación curvas of force of reacción to the suelo; Métricas biomechanical: force pico of despegue (fase concéntrica maximal antes of the despegue) and force pico of aterrizaje (force maximal during fase of aterrizaje)	objective monitoring of inter-limb asymmetries as indicators of functional status and non-contact injury risk (especially knee/ankle).	(r~0.500-0.800); Asymmetry force despegue: CMJ significativamente greater que SLJ (global: 7.2% vs. 1.0%; volleyball: 7.2% vs. 1.1%; baloncesto: 7.1% vs. 0.9%) with efectos grandes (r~0.800); Asymmetry force aterrizaje: patrón inverso with SLJ significativamente greater que CMJ (global: 6.8% vs. 3.3%
42	Pisa,	Internal	Brazil	Triple	Systematic	range	Mapeo systematic of	Síntesis of	Herramientas validadas

No.	Authors (Year)	Article title	Country	Primary aim	Sample	Age	Technology/AI	Load monitoring domain	Key findings
	M.F., Zecchin, A.M., Gomes, L.G., Norberto, M.S., Puggina, E.F. (2022) [32]	load in male professional volleyball: A systematic review	(Universidade of São Paulo - Escola of Educação Física, Ribeirão Preto Medical School)	propósito systematic: (1) Sintetizar valores of load of training interna (IL) presentes in the literatura on volleyball masculino profesional; (2) Verificar the aplicabilidad of diferentes herramientas to cuantificación and monitoreo of variables of IL during sessions of training and matches;	review (PRISMA): 26 studies including 381 professional male volleyball athletes; search period January 2000–May 2020 (after FIVB 1998 rule changes, including libero introduction and scoring-system updates).	heterogéneo según studies included (review sistemática multi-study with criterios profesionales)	herramientas of monitoreo no invasivo of internal load: Métricas subjetivas predominantes: Escala of esfuerzo percibido (RPE Borg CR-10: 0-10, Borg 6-20), RPE of session (sRPE = RPE × duración), questionnaires of well-being and recovery; Métricas objetivas complementarias: heart rate (FC), TRIMP (Training Impulse métodos Edwards/Stagno)	aplicabilidad of herramientas of internal load: sRPE as herramienta central: more utilizada and efectiva by bajo costo, facilidad aplicación and confiabilidad; valores range 51.92-627 AU with cinética ondulante intra e inter-weekly; Patrones temporales identificados: TWTL presenta distribución ondulante (período preparatory > competitivo), loads more altas 5 días antes o 1-2 días post-partido	predominantes: RPE and sRPE identificadas as métodos more utilizados and efectivos to cuantificación IL by confiabilidad, bajo costo and facilidad of aplicación; Valores of referencia specific: RPE training típico 4-7 (CR-10), sRPE range 51.92-627 AU, TWTL mean ~3185 AU with variación estacional sig.; Patrones temporales críticos
43	Taylor J.B., Barnes H.C., Gombatto S.P., Greenwood	Quantifying External Load and Injury Occurrence in Women's	United States (High Point University, San Diego State University, University	examine the demandas of jump (external load) and patrones of injuries during A	16 volleyball players female NCAA División I (9 atacantes -	19.6 ± 1.1 years (range 18-21); height: 1.78 ± 0.07 m; masa: 75.9 ± 9.3 kg	Technology no invasiva: inertial measurement unit (IMU) VERT Classic with accelerometer, transmisión Bluetooth A tablet, aplicación "Vert Coach" to seguimiento in real time. Validada to	Monitoreo longitudinal comprehensivo: Conteo of jumps (JC), height mean (JH), height of 5 jumps máximos (JH5), jump load	Demandas temporales: preseason with greater loads of training (JC, JL) vs. no-conferencia, conferencia, posseason (p<0.001). performance mejoró progresivamente (JH, JH5 superiores in

No.	Authors (Year)	Article title	Country	Primary aim	Sample	Age	Technology/AI	Load monitoring domain	Key findings
	D., Ford K.R. (2022) [27]	Collegiate Volleyball players Across A Competitive Season	of Memphis)	season completa of women's volleyball collegiate; analyze relaciones between load of training and ocurrencia of injuries by fase of season, tipo of session and tiempo of juego	3 bloqueador as middle blockers, 4 atacantes externas, 2 atacantes opuestas-, 5 defensoras -3 especialist as defensivas, 2 liberos-, 2 armadoras). 100% población femenina aim		count and height of jumps in volleyball. Componente algorithmic: algorithms MICE (Multiple Imputation by Chained Equations) to imputación predictiva of data faltantes (15)	novedosa (JL = JC × JH × masa corporal). Seguimiento temporal: 17 weeks completas, 58 prácticas tácticas, 33 matches totales. assessment multidimensional: questionnaire OSLO modificado weekly to injuries by sobreuso, registro of injuries with pérdida of tiempo	conferencia vs. preseason, $p \leq 0.01$). difference contextuales: without differences globales práctica vs. partido
44	Patti A, Gervasi M, Giustino V, Figlioli F, Canzone A, Drid P, Thomas E, Meswithouta G, Vicari	the Influence of Ankle Mobility and Foot Stability on Jumping Ability and Landing Mechanics: A Cross-Sectional Study	Italy (Universidad of Palermo, Universidad of Urbino Carlo Bo), Serbia (Universidad of Novi Sad)	examine the conexión between the range of motion of the tobillo (ROM) and the estabilidad of aterrizaje after jumps; evaluate the correlation between ROM of the tobillo and	62 players amateur of volleyball (37 women, 25 men; 59.67% women)	16.5 ± 4.25 years (height: 166 ± 11.4 cm; peso: 61.6 ± 13.7 kg; pie dominante: derecho *n* = 55, izquierdo *n* = 7)	Sensor inercial: BEYOND Inertial Bluetooth (up to 1000 Hz; accelerometer ±2G-±16G, gyroscope ±200°/s-±2000°/s, magnetometer ±4000μT) to ROM tobillo (dorsiflexión, flexión plantar, eversión, inversión) with parameters: range motion, velocity angular, índice fluidez. Sistema óptico: Microgate to CMJ	assessment multidimensional of the estado neuromuscular and control postural through: CMJ as indicador capacity explosiva and readiness; ROM tobillo as factor biomecánico crítico to performance and prevención injuries; Tarea	ROM–performance associations: right-ankle dorsiflexion ROM vs CMJ, r = 0.81 ($p < 0.001$; strong); left-ankle dorsiflexion ROM vs CMJ, r = 0.46 ($p < 0.001$; moderate). Multiple regression models predicted CMJ performance uwithoutg ROM variables.

No.	Authors (Year)	Article title	Country	Primary aim	Sample	Age	Technology/AI	Load monitoring domain	Key findings
	DSS, Palma A, Bianco A (2024) [56]			height of jump in CMJ; analyze the estabilometría during motions specific of jump through A tarea personalizable adaptada A características individuales			(accuracy 1/1000s, tiempos vuelo/contacto)	específica jump-aterrizaje personalizada (distancia=½height sujeto, obstáculo=70% CMJ individual) to caracterizar estabilidad dinámica post-aterrizaje during 20s	
45	Coyne JOC, Coutts AJ, Newton RU, Haff GG (2021) [52]	the Influence of Mental Fatigue on Sessional Ratings of Perceived Exertion in Elite Open and Closed Skill Sports athletes	Multicéntrico: Australia (Edith Cowan University), United States (UFC Performance Institute), United Kingdom (University of Salford)	examine the influencia of the fatigue mental on the calificaciones sesionales of esfuerzo percibido (sRPE) during A week completa of training in athletes of elite of deportes of habilidad abierta (OS: baloncesto, volleyball)	Group OS (*n* = 27 after exclusiones): 21 athletes female volleyball + 11 athletes female baloncesto (group OS exclusivamente female). Group CS (*n* = 28): 27 athletes halterofilia (13♂, 14♀) + 4 athletes	women's volleyball: 26.4 ± 3.6 years (187.5 ± 6.8 cm, 75.5 ± 6.7 kg); Baloncesto female: 28 ± 3.7 years (185.9 ± 10.1 cm, 78.7 ± 11.5 kg); Halterofilia: 27.0 ± 3.2 years; Atletismo: 24.1 ± 1.3 years	Mental fatigue VAS: validated 100-mm visual analogue scales (Cronbach's α = 0.91–0.98) administered within 30 min post-training. sRPE: Borg CR-10 (CV = 8.5%). Training load: sRPE × duration (sRPE-TL).	Monitoreo longitudinal integrado during week completa típica (8-10 sessions técnicas + 3-4 no técnicas, 409 sessions totales): sRPE as medida global integrando respuestas físicas/psicológicas/cognitivas to the training; fatigue mental VAS as biomarker independiente of the estado psicobiológico; analysis	Correlaciones fatigue mental-sRPE: Group OS: r=0.23, p=0.001 (correlation pequeña pero sig.); Group CS: r=-0.07, p=0.38 (no sig.). difference between groups: OS > CS in fatigue mental individual (p=0.001, f^2=0.089) and weekly (p<0.001, g=1.29, efecto grande). Paradoja cognitiva-perceptual: OS greater fatigue mental pero lower sRPE medio by session vs CS (4.1±1.3 vs 4.8±2.1, p=0.01). Efectos predictive

No.	Authors (Year)	Article title	Country	Primary aim	Sample	Age	Technology/AI	Load monitoring domain	Key findings
				vs. cerrada (CS: halterofilia, atletismo); explorar differences in leveles of fatigue mental between groups OS and CS	masculinos atletismo. level: 78% OS and 97% CS with experience olímpica/ mundial, teams nacionales senior			diferencial deportes OS vs CS to caracterización específica of respuestas A the load	
46	Uslu S, Abazović E, Čaušević D, Mahmutović I, Riza B (2021) [57]	the Relationship Between Isokinetic Strength and Jump Performance in Elite Female Volleyball players	Multicéntrico: Turkey (Gazi University), Bosnia and Herzegovina (University of Sarajevo)	Evaluate associations between knee extensor/flexor strength and vertical jump performance in professional female volleyball players; assess whether strength–jump correlations differ across strength-level groups.	41 players profesionales of women's volleyball (100% women)	20.37 ± 4.2 years (height: 183.3 ± 8.5 cm; peso: 72.2 ± 12.9 kg)	Dinamometría isocinética: Isomed 2000 (Ferstl, Germany) to assessment force concéntrica extensores/flexores knee A 60°/s (10 repeticiones), measurement bilateral with estabilización completa and protocolo estandarizado. Plataforma of force: Newtest (Finland) portable to CMJ with protocolo Bosco et to the. (1983), manos in hips, 3 intentos máximos with 2min recovery	assessment capacityes neuromusculares through: force isocinética as indicador específico of the estado of the sistemas extensores/flexores of knee bajo condiciones controladas; CMJ as medida funcional integrada of power explosiva and readiness neuromuscular; analysis diferencial by dominancia to detección asymmetrys	Correlaciones force-performance significativas: Extensores knee dominante-CMJ: r=0.596, p<0.01; Flexores knee dominante-CMJ: r=0.559, p<0.01; Correlaciones superiores in pierna no-dominante: Extensores no-dominante-CMJ: r=0.673, p<0.01; Flexores no-dominante-CMJ: r=0.626, p<0.01. Analysis by groups of force: Group débil (PT/BW<2): correlaciones significativas solo with pierna no-dominante
47	Bertozz	the role of	Italy	examine the	45 players	range 12.5-	Plataformas of force	assessment	MO as predictor superior:

No.	Authors (Year)	Article title	Country	Primary aim	Sample	Age	Technology/AI	Load monitoring domain	Key findings
	i F, Brunetti C, Maver P, Galli M, Tarabini M (2025) [20]	age and maturation on jump performance and postural control in female adolescent volleyball players over A season	(Politecnico di Milano)	efecto of the Age cronológica and maduración biológica (evaluada as distancia of the pico of velocity of crecimiento usando maturity offset - MO) on capacityes of jump vertical/pliométrico and control postural in players adolescent of women's volleyball A través of diferentes categorías of Age during A season competitiva	adolescent of women's volleyball (100% women) of the mismo club. Distribución: U13 (*n* = 10), U14 (*n* = 11), U16 (*n* = 12), U18 (*n* = 12)	17 years. by categorías (preseason): U13: 12.47±0.45, U14: 13.06±0.48, U16: 14.99±0.55, U18: 16.55±0.57 years	piezoeléctricas: Dos Kistler 9260AA (600×500mm, range 0-5kN) with software BioWare v5.4.9.0, muestreo 1000Hz (jumps) and 100Hz (equilibrio estático). Procesamiento: MATLAB R2024a with filtros Butterworth bidireccionales (50Hz force, 10Hz CoP). Variables CMJ: height, momentum, power pico, impulso concéntrico/excéntrico	longitudinal readiness neuromuscular: Seguimiento of capacityes explosivas (CMJ) and reactivas (DJ) as indicadores of the estado neuromuscular during the season; Monitoreo maduración biológica: MO as factor modulador crítico of the performance independiente of Age cronológica to individualización of loads; Control postural: assessment estabilidad as componente of the readiness funcional	association positiva sig. with power pico (R^2=0.48), momentum jump (R^2=0.57), impulso concéntrico (R^2=0.31), impulso excéntrico (R^2=0.57) in CMJ (todos p<0.001). Especificidad of evaluaciones: CMJ mostró efectos significativos of session (p=0.010, η^2p=0.29) and MO (p=0.025, η^2p=0.49)
48	Giatsis G, Panoutsakopoulos V, Frese	Vertical Jump Kinetic Parameters on Sand and	Grecia (Aristotle University of Thessaloniki),	investigate the possible differences in the performance and	11 players young of volleyball playa female with	21.2 ± 2.3 years (height: 1.74 ± 0.04 m; peso: 64.1 ± 3.5 kg).	Plataforma of force: AMTI OR6-5-1 (500 Hz) to forces of reacción of the suelo (GRF). Cámara alta velocity: Redlake Motionscope PCI 1S	Caracterización biomechanical integral: assessment of respuestas neuromusculares	Efectos principales of superficie: hJUMP lower in SJS vs RJS (p<0.05); tUz significativamente lower in SJS (ηp^2=0.433, efecto grande) - athletes

No.	Authors (Year)	Article title	Country	Primary aim	Sample	Age	Technology/AI	Load monitoring domain	Key findings
	C, Kollias AI (2023) [43]	Rigid Surfaces in Young Female Volleyball players with A Combined Background in Indoor and Beach Volleyball	Germany (University Stuttgart)	parameters biomecánicos of tests diagnósticas comunes of jump vertical ejecutadas in superficies rígidas (RJS) and of arena (SJS) in players young of women's volleyball with experience combined indoor/playa	experience combined (7 miembros of selección nacional, 4 with participación in competiciones internacionales greater, 4 level nacional, 2 level collegiate)	Criterios inclusión: Participación in torneos oficiales BV últimos 5 years, training systematic indoor >10h/week invierno previo	(250 fps) to verificación interacción pie-arena. Setup arena: Foso madera (0.59×0.63m superior, 0.46×0.50m inferior) with 112.12 kg arena (profundidad 0.31m) cumpliendo estándares FIVB/ASTM	específicas A diferentes superficies of training through 4 tipos of jump (SQJ, CMJA, CMJF, DJ40) with analysis of parameters temporales (tC, tFz, tUz, tPMAX), espaciales/cinemáticos (hJUMP, SDOWN, UzMAX, hDROP) and cinéticos (FzMAX, RFD, PMAX)	alcanzan velocity pico CoM more rápido in arena. Ganancia SSC crítica: Significativamente greater in SJS (15.4%) vs RJS (7.5%) ($p=0.031$, $d=0.93$, efecto grande), indicando mejor aprovechamiento of the pre-estiramiento in arena. Parámetros cinéticos conservados
49	Ungureanu AN, Brustio PR, Boccia G, Rainoldi A, Lupo C (2021) [48]	Effects of Presession Well-Being Perception on Internal Training Load in Female Volleyball players	Italy	evaluate si the internal load of training (ITL: métodos Edwards basado in HR and session-RPE) es afectada by the percepción of well-	*n* = 12 players elite women's volleyball Serie A2 italiana (100% women): 3 atacantes, 2 liberos, 3 middle blockers, 2 opuestas, 2 colocadora	22 ± 4 years; height: 1.80 ± 0.06 m; peso: 74.1 ± 4.3 kg	Monitoreo no invasivo validado: Polar H10 (registro HR cada 1s, transmisión Bluetooth A iPad Air); método Edwards HR-based (5 zonas %HRmax with coeficientes 1-5 sumados); escala CR-10 Borg modificada Foster to RPE 20 min post-session; Índice Hooper to well-being pre-session (sleep, stress, fatigue, DOMS, escala 1-	Monitoreo multidimensional systematic: ITL cuantificada through Edwards HR-based (tiempo acumulado zonas HR × coeficientes) and session-RPE (RPE × duración session); assessment well-being pre-session	Hallazgo metodológico central: fatigue percibida pre-session influenció significativamente ITL según session-RPE ($\beta=32.97$, $p=0.032$, $d=0.13$) pero NOT según método Edwards HR, demostrando greater sensibilidad percepción subjetiva vs respuesta fisiológica objetiva in deportes intermitentes explosivos. Efectos Age: correlation inversa with

No.	Authors (Year)	Article title	Country	Primary aim	Sample	Age	Technology/AI	Load monitoring domain	Key findings
				being pre-session, Age and posición táctica in players elite of women's volleyball Serie A2	s. Criterios inclusión: ≥8 years experience volleyball, ≥2 years training 4-7 sessions/week 90-180 min, >80% participación weekly		7)	through Índice Hooper multidimensional; 290 sessions individuales monitoreadas in 32 sessions team (duración mean 1:36:12 ± 0:22:24); analysis diferencial by posición táctica and Age	Edwards (β=-4.04, p<0.001, d=-0.28) and directa with session-RPE (β=5.77, p=0.027, d=0
50	Ungureanu AN, Lupo C, Boccia G, Brustio PR (2021) [49]	Internal Training Load Affects Day-After-Pretraining Perceived Fatigue in Female Volleyball players	Italy	evaluate si the internal load (session-RPE and método Edwards basado in HR) and externa (jumps) of training afectan the percepción of well-being pre-session to the día siguiente (+22h), según Age and posición táctica, in players elite	*n* = 10 players elite women's volleyball Serie A2 italiana (100% women): 3 atacantes, 3 middle blockers, 2 opuestas, 2 colocadoras (without liberos). Criterios inclusión: ≥8 years experience volleyball, ≥2 years training 4-7	23 ± 4 years; height: 1.82 ± 0.04 m; peso: 73.2 ± 4.9 kg	Monitoreo no invasivo integrado: Polar H10 (registro HR cada 1s, transmisión Bluetooth A iPad Air); método Edwards HR-based (5 zonas %HRmax estimado 220-Age with coeficientes 1-5); analysis video systematic jumps (Canon Legria HF R46, height ≥5m, 6 observadores, ICC=0.9, CV=5.6%); escala CR-10 Borg modificada Foster to RPE 20 min post-session	Focus temporal innovador: ITL cuantificada through Edwards (tiempo acumulado zonas HR × coeficientes) and session-RPE (RPE × duración); ETL through count systematic jumps by analysis video (ambos pies despegue, independiente intensidad/habilidad); assessment well-being Hooper +22h post-load; 115 sessions individuales in 13 sessions team	Hallazgo temporal central: Solo ITL (no ETL-jumps) afectó well-being +22h with efectos opposites: session-RPE correlacionó positivamente with fatigue percibida (β=0.001, p<0.001, d=0.54) e Índice Hooper (β=0.003, p=0.005, d=0.21), while Edwards correlacionó negativamente with fatigue (β=-0.0018, p=0.042, d=-0.21) e Índice Hooper (β=-0.007, p=0.041, d=-0.25)

No.	Authors (Year)	Article title	Country	Primary aim	Sample	Age	Technology/AI	Load monitoring domain	Key findings
				of women's volleyball Serie A2	sessions/week 90-180 min, participación 2 sessions consecutivas 24h			específicas (duración 1:41:01 ± 0:07:52)	
51	Rabbani M, Agha-Alinejad H, Gharakhanlou R, Rabbani A, Flatt AA (2021) [51]	Monitoring training in women's volleyball: Supine or seated heart rate variability?	Irán (institución líder), colaboración United States	examine changes in HRV in rest, heart rate ejercicio submaximal (HRex), recovery cardiac (HRR), height jump contramotion (CMJ), well-being perceptual (Hooper's Index) and internal load (sRPE) during fields training preparatory in players elite women's volleyball; determine which posición	*n* = 13 players women's volleyball team nacional iraní (100% women); todas aprobadas médicamente to participación deportiva irrestricta; compliance alta: HRV 95%, HRex/HRR 90%, CMJ 97%, Hooper/sRPE 100%	25.8 ± 3.0 years; height: 178.1 ± 6.7 cm; peso: 69.7 ± 7.6 kg	Monitoreo no invasivo validado: Sensor HR Bluetooth Polar H7 (validado vs. ECG: differences triviales, r≥0.99, límites concordancia estrechos) pareado with aplicación Elite HRV™ to LnRMSSD in positions supina/sentada randomizadas (60s measurement after 60s estabilización, respiración espontánea, post-despertar/micción); proceswithoutg data R-R with software Kubios HRV (filtro paso-bajo estándar)	Monitoreo multidimensional integrado: HRV diaria calculando LnRMSSDM (mean weekly) and LnRMSSDCV (coeficiente variación diaria) mínimo 3 registros/field; evaluaciones fitness cardiorrespiratoria (HRex mean últimos 30s, HRR latidos recuperados 1 min) and neuromuscular (CMJ 3 intentos máximos, 1 min descanso) inicio cada field; well-being subjetivo diario post-despertar; internal load session-RPE	Efectos temporales significativos field 3: LnRMSSDCV sentada (p=0.029, ES moderado), HRex (p=0.002, ES moderado), CMJ (p=0.001, ES moderado), sRPE (p=0.006, ES moderado) aumentaron significativamente vs. field 2, revirtiendo A valores similar field 2 in field 4, indicando patrón load-recovery. Superioridad metodológica HRV sentada

No.	Authors (Year)	Article title	Country	Primary aim	Sample	Age	Technology/AI	Load monitoring domain	Key findings
				measurement HRV (supina vs. sentada) provides greater associations with markers adaptation training					
52	Pelzer T, Schmidt M, Jaitner T, Pfeiffer M (2020) [23]	External training load and the effects on training response following three different training sessions in young elite beach volleyball players	Germany (Olympic Training Center Rhineland-Palatinate/ Saarland, TU Dortmund University, Johannes-Gutenberg University Mainz)	analyze external load of training (ETL) and efectos on respuesta of training in diferentes configuraciones of volleyball playa; evaluate interacción between volumen of jumps and otros tipos of acciones with markers of respuesta fisiológica	*n* = 7 athletes of elite youth (18.9±1.3 years, 185.2±7 cm, 75.9±10.4 kg, 5 women/2 men = 71.4% femenina), level nacional of categorías U19 and U23	18.9±1.3 years (athletes of elite youth with experience competitiva nacional)	Non-invasive technologies: Vert IMU sensor (model #CC2020; ICC = 0.97 for spike and 0.96 for block) for jump count and height; Optojump Next for CMJ; synchronized video analysis (inter-rater kappa 0.94–0.98); perceptual scales.	external load: Volumen of jumps by session (SJV), Load Index (energía cinética × frecuencia), distribución tipos of acciones (jumps, buceos, recepciones, defensas); internal load: sRPE, DOMS (escala 0-10), CK sérico, CMJ pre-post; Design: 3 tipos of session with proporciones diferenciadas of acciones	Hallazgo paradigmático: the volumen of jumps NOT predice respuesta of training. Sessions with greater frecuencia of buceos and acciones of recovery rápida (Tipo C: 39 jumps totales) generaron greater respuesta interna (DOMS, CK, sRPE=689±158 AU) que sessions with alto volumen of jumps (Tipo A: 118 jumps, sRPE=595±197 AU). Load Index mostró relationship inversa with markers of fatigue
53	Bouzigues	Quantification of	France (Ecole	1) evaluate si the STR	*n* = 19 athletes	17.7±1.3 years	Non-invasive technologies: G-Vert	external load: 140,321 jumps	Best-performing model: Random Forest

No.	Authors (Year)	Article title	Country	Primary aim	Sample	Age	Technology/AI	Load monitoring domain	Key findings
	Théo, Robin Candau, Sami Äyrämö, Olivier Maurelli, Jacques Prioux (2025) [14]	workload and characterisation of key performance factors in elite adolescent female volleyball players uwithout g machine learning	Normale Supérieure of Rennes, INRAe Center of Montpellier, University of Rennes 2, French Federation of Volleyball), Finlandia (University of Jyväskylä) - colaboración franco-finlandesa	score provides mejor cuantificación of load que métodos tradicionales to explicación/prediction of performance of jump; (2) identify si the training of alta intensidad es the principal contribuyente to the performance of jump; (3) determine si models no-lineares son more efectivos que lineares to explicar/predecir performance of jump	female of elite youth (17.7±1.3 years, 73.5±7.8 kg, 182.4±4.3 cm), centro nacional of training francés, liga francesa of maximal level. Composición positional: armadoras (*n* = 4), atacantes externas (*n* = 8), middle blockers (*n* = 5), opuestas (*n* = 2). Exclusión: liberos by especificidad positional diferenciada	(athletes of elite youth with potencial of selección nacional francesa)	accelerometer (Matsport Training, Grenoble) validated for volleyball; Mycoach Pro mobile app for multidimensional perceptual scales. ML algorithms included Elastic Net and other models.	totales monitoreados during 190 días, height e intensidad by jump (5 zonas: Z1=0-20%, Z2=20-40%, Z3=40-60%, Z4=60-80%, Z5=80-100%), workload exponencial individualizado, STR score innovador with constantes temporales τ1=5días (efectos positivos duraderos), τ2=1día (fatigue inmediata)	outperformed Elastic Net and ANN (R^2 = 0.64; MAE = 3.5 ± 0.4), indicating superiority of non-linear approaches. Key predictors included well-being/strain composite indicators and jump-load features.
